# A woman's lifetime risk disparities in maternal mortality in Ethiopia

**DOI:** 10.1002/puh2.56

**Published:** 2023-01-13

**Authors:** Moges Tadesse Borde

**Affiliations:** ^1^ School of Public Health, College of Health Sciences and Medicine Dilla University Dilla Ethiopia

**Keywords:** death, difference, Ethiopia, lifespan, woman

## Abstract

**Background:**

In Ethiopia, a woman's lifetime risk (chance) of dying from maternal causes over a woman's reproductive lifespan continues to be a public health challenge. However, detailed evidence is scarce. This study aimed to assess woman's lifetime risk disparities in maternal mortality.

**Methods:**

National data from the Ethiopian Demographic and Health Survey from 2000 to 2016 were used. To determine a woman's lifetime risk of dying, first, the maternal mortality ratio was calculated. Then, a woman's lifetime risk of dying was calculated. Four policy‐relevant socio‐economic factors were assessed: residence, education, regional states and economic status. To assess a woman's lifetime risk disparities in maternal mortality, the rate ratio (*R*) was used as per the World Health Organization's Health Equity Assessment Toolkit and software.

**Results:**

From 61,610 respondents, 795 deaths were reported. A woman's lifetime risk of dying nearly halved from 4.6% in 2000 (1 in 22 women) to 1.9% in 2016 (1 in 52 women). In 2016, the highest woman's lifetime risk of dying was observed among rural women (1 in 111 women), in Oromiya regional state (1 in 71 women), women with secondary education (1 in 82 women) and women with middle income (1 in 77 women). The socioeconomic factors that drive a woman's lifetime risk disparities in maternal mortality include residence (1.3‐times higher among rural women), regional state (2‐times higher among women in Oromiya regional state), education (6‐times higher among women with lower educational status), and economic status (1.3‐times higher among the poorest women).

**Conclusion:**

Ethiopian women experienced a high lifetime risk of maternal mortality which varies across socioeconomic groups. Therefore, equity‐oriented maternal healthcare is recommended to reduce the effects of structural inequities, such as poverty, and the inequitable distribution of the socioeconomic factors of maternal health that sustain a woman's lifetime risk disparities in maternal mortality.

## INTRODUCTION

Ethiopia is one of the signatories of the Sustainable Development Goals (SDGs) which targets to reduce the Maternal mortality ratio (MMR) to 70 or fewer per 100,000 live births by 2030 [1]. As the MMR in Ethiopia declined only by half from 872 to 412 per 100,000 live births from 2000 to 2016 [2], a woman's lifetime risk (LTR) disparities in maternal mortality due to maternal causes over a woman's reproductive lifespan continue to be a public health challenge [3]. Therefore, an in‐depth and more informative measurement of maternal mortality is required that has gone beyond the rate and/or ratio of maternal mortality, which are often used [4].

Measurement of a woman's LTR (chance) of dying could be an alternative and reliable measurement of maternal mortality and is highly recommended as a summary measure of maternal health and is used as a reflection of the whole national health‐care system [4]. Yet, MMR and/or MM rate (maternal mortality rate) are being used as a measurement of maternal mortality other than a woman's LTR of dying [4]. MMR and/or MM rate measures only the risk of maternal death once a woman has become pregnant. In contrast, a woman's LTR of dying refers to the probability of maternal mortality due to maternal causes over a woman's reproductive lifespan and measures the cumulative loss of a woman's life taking into account both the probability of becoming pregnant and the chance of dying as a result of that pregnancy [4].

The measurement of a woman's LTR of dying can be derived using either the MMR or MM rate, assuming that neither the MMR nor the MM rate is constant over the woman's reproductive lifespan. The relationship between MMR and LTR yields an estimate of a woman's LTR of dying that takes into account competing causes of death [5]. Trends in maternal mortality could also be interpreted in light of a woman's LTR of dying per woman [6]. The importance of measuring the socioeconomic factors that drive a woman's LTR disparities in maternal mortality was that it reflects at least two underlying concepts: the fraction of female infants who would die eventually from birth until menopause; and the fraction of adolescent girls who would die in their lifetime from maternal causes [4].

In 2016, according to Ethiopian Demographic and Health Survey (EDHS), the prevalence of a woman's LTR of dying in Ethiopia was 1.9% (1 in 52 women), which was much higher than in other countries [2]. Yet, in 2018, a study conducted in Kersa district, Eastern Ethiopia, showed that the prevalence of a woman's LTR of dying was 4.3% (1 in 23 women) [7]. In other words, in 2019, according to a report by the World Health Organization (WHO), the prevalence of a woman's LTR of dying in Ethiopia was 1.8% (1 in 55 women); however, it was better than the average in sub‐Saharan African countries, 2.7% (1 in 37 women); and in Africa as a whole, 2.2% (1 in 45 women); but, it was the worst when compared to the global average, 0.5% (1 in 190 women) [3]. In 2022 also, according to a report by the World Bank, the prevalence of a woman's LTR of dying in Ethiopia was 1.5% (1 in 67 women), and Ethiopia was ranked number 30 in a woman's LTR of dying out of 185 countries in the world. In this regard, Chad was ranked number one in a woman's LTR of dying with 6.6% (1 in 15 women). In contrast, the United Arab Emirates and some European and Scandinavian countries were ranked number 178 up to 185 in a woman's LTR of dying with 0% [8].

The prevalence of a woman's LTR of dying also varies with socioeconomic disparities in maternal mortality [2]. Disparities in maternal mortality are one of the maternal health *disparities* that refer to differences in *health* and *health*care among women and/or geographic areas [9]. Besides, it is believed that using the measurement of a woman's LTR disparities in maternal mortality yields more accurate estimates of maternal mortality than MMR [4], which could also be used as an indicator of maternal mortality that could be averted, serve as a key economic indicator of maternal health in a nation and could be useful as metric for understanding the impact of the causes of maternal mortality, and could progress in reducing maternal mortality [10]. Moreover, assessing the socioeconomic factors that drive a woman's LTR disparities in maternal mortality could help to inform policymakers and could have a significant role in achieving SDG‐3 as well as the goals of Universal Health Coverage.

The rationale of this study is that detailed evidence of a woman's LTR disparities in maternal mortality is scarce, and little is known about whether a woman's LTR disparities in maternal mortality differ among geographic areas or regional states in Ethiopia. A woman's LTR disparities in maternal mortality are a very important subject as health equity and ways for improvement remain significant concerns in public health. This study has also taken an in‐depth view into maternal mortality as considerations have gone beyond just the rate and ratio of maternal mortality often used. Therefore, the current study can contribute to the available evidence by assessing a woman's LTR disparities in maternal mortality using policy‐relevant socioeconomic factors, as per the recommendation of the WHO's than the traditional methods [11]. Nonetheless, a limited number of studies attempted to assess the socioeconomic factors that drive a woman's LTR disparities in maternal mortality, except a study on trends and causes of maternal mortality in 2017 [12], and a study that indicated the high burden of maternal mortality in rural south Ethiopia [13].

To fill the research gap, this study aimed to assess a woman's LTR disparities in maternal mortality and to identify the socioeconomic factors that fuel these disparities at the national and regional state levels in Ethiopia. Two research questions were designed: What is the status of a woman's LTR disparities in maternal mortality in Ethiopia? And, whether there were residence, regional states, education and wealth‐based woman's LTR disparities in maternal mortality in Ethiopia. I hypothesize that a woman's LTR disparities in maternal mortality in Ethiopia could be more pro‐poor, pro‐rural areas, higher among women with lower educational status, and among women who were living in the least developed regional states.

## METHODS

### Study design and data sources

Nationally representative cross‐sectional EDHS from 2000 to 2016 were used to compute the cumulative LTR of dying in the country. The weighted samples taken at 5‐year intervals in 2000 [14], 2005 [15], 2011 [16] and 2016 [2] were used. All EDHS were conducted using a similar approach: sample design and selection, survey methodology and ethical approval [17]. For example, the sampling frame used for surveys was a frame of all census enumeration areas (EAs). The census frame is a complete list of the EAs which is a geographic area covering an average of 131 households per EA. The EDHS sample was stratified and selected in two stages. Each region was stratified into urban and rural areas. Samples of EAs were selected independently in each stratum in two stages. Implicit stratification and proportional allocation were achieved at each of the lower administrative levels by sorting the sampling frame within each sampling stratum before sample selection, according to administrative units in different levels, and by using a probability proportional to size selection at the first stage of sampling. The EDHS also utilizes standardized and comparable questionnaires, measurement techniques and well‐trained interviewers [17].

### Study setting

Ethiopia is a country located in the horn of Africa with over 109 million inhabitants [18]. Of the current population, 51% are women, 46% fall between 1 and 14 years, 51% are between 15 and 64 years, and 3% are over 65‐year old. Approximately 17% of the population is estimated to live in urban areas. The overall life expectancy of the population is currently 65 years [19]. In Ethiopia, there are nine regional states and two city councils that are classified into three categories: emerging regions, that is Afar, Somali, Benishangul‐Gumuz and Gambella regional states; developed regions, that is Amhara, Oromiya, Harari, Southern Nations Nationalities and People's Region (SNNPR) and Tigray regional states; and two city councils, that is Addis Ababa and Dire Dawa councils [20]. A total of 13.1 million people in Ethiopia are living in emerging regions that are scattered pastoralists and semi‐pastoral populations with extreme poverty and limited access to healthcare [21]. The Ethiopian economy is dominated by the agriculture and service sectors, and agriculture remains the Ethiopian economy's most important sector [19]. Despite the significant reduction in the number of people living in severe poverty, and showing the fastest economic growth over the last decade [18], Ethiopia is still one of the poorest nations in the world, with an average per capita Gross Domestic Product of US$934 in 2020 [22]. According to the Human Development Index, Ethiopia ranks 173 out of 189 countries, in terms of socioeconomic dimensions, that is life expectancy, literacy and education [23].

In Ethiopia, the health‐care system is organized into a three‐tier system based on the type of healthcare provided (i.e. primary, secondary and tertiary) [24]. The primary tier system is the first level and is named a primary health‐care unit, and there is one health centre with five health posts and one primary hospital. The health centre serves about 15,000–25,000 people in rural areas and about 40,000 people in urban areas, meanwhile each health post serves 3000–5000 people in rural areas, and the primary hospital serves 60,000–100,000 people. The secondary tier system comprises one general hospital that serves about 1–1.5 million people, and the tertiary tier system also comprises one specialized hospital that serves about 3.5–5 million people. The national referral system is done from the community to the health post, to the health centres, to hospitals and within hospitals and vice versa. It also involves not only direct patient care but also other support services such as transport and communication [24].

In this study, maternal mortality is defined as the death of a woman during pregnancy, childbirth or the postpartum period (i.e. in the first 42 days after birth) irrespective of the duration and site of the pregnancy, from any cause related to or aggravated by the pregnancy or its management, but excludes deaths due to accidents or incidental causes [25]. A woman's LTR of dying refers to the probability that a 15‐year‐old woman will eventually die due to maternal causes over a woman's reproductive lifetime [4]. The term disparity refers to differences among comparison groups. Disparity often mixes ideas of inequality and inequity [26].

### Study variables

The primary outcome variable was a woman's LTR disparities in maternal mortality, but the secondary outcome variable was a woman's LTR of dying. Four policy‐relevant socioeconomic factors or predictor variables were assessed, namely residence, geographical areas (regional areas), education and economic status. For educational status also, four categories were used, that is no education, primary, secondary and higher education. For residence, two categories were used, that is urban and rural. For the regional states also, the nine regional states and the two city councils were used, that is Tigray, Amahara, Oromiya, SNNPR, Somali, Harar, Afar, Benishangul‐Gumuz and Gambella regional states, and Addis Ababa and Dire Dawa councils.

In EDHS, the direct sisterhood method was used to estimate maternal mortality using survived sisters as respondents, sisters who died, and the number of years before the survey that the sister(s) died. For each sister who died at age 12 or over, three questions were used to determine if the death was maternity related, that is “Was pregnant when she died?”, “Did she die within two months of the birth of a child or pregnancy termination?” whether the sister died within the termination of a pregnancy or childbirth. To construct the wealth index also, EDHS used information on household assets ownership, housing and environmental conditions [2].

As the primary outcome variable was the adverse health outcome, that is for the socioeconomic‐driven LTR disparities in maternal mortality, two types of data were required: data on “adverse health outcome indicators” that was woman's maternal mortality experience and data on “dimensions of disparity” that allow women to be grouped into their socioeconomic status, namely residence, geographic locations (regions), education and wealth [27].

### Data analysis

The “LTR of dying” was approximated by [LTR  =  1 − (1 − MMR)]. To obtain a woman's LTR of dying, I first calculated the MMR by dividing the number of maternal mortality by the total number of reproductive‐aged women [7] (Supporting Information [Link], [Link], [Link], [Link]).

A woman's LTR disparities in maternal mortality by socioeconomic factors were described by the rate ratio [28]. Rate ratio (*R*) is a simple, relative and unweighted measure of disparity in a woman's LTR s of dying that shows the relative disparity between two extreme subgroups [27]. Therefore, as the outcome variable was an adverse outcome, for non‐ordered dimensions, such as the place of residence, the urban was selected as a reference group, and the calculation of *R* = *R*_rural_/*R*_urban_. For non‐ordered geographical areas (regional states), the nine regional states and two city councils were considered. Addis Ababa was selected as a reference group and the calculation of *R* = *R*_disadvantaged subgroup_/*R*_reference subgroup_, whereas for ordered dimensions, such as wealth quintile and education, the calculation of *R* = *R*_disadvantaged subgroup_/*R*_advantaged subgroup_ [20]. For example, the poorest quintile was divided by the richest quintile, and no education subgroup was divided by the secondary education subgroup [29].

An exploratory data analysis was carried out to ascertain maternal mortality and the selected socioeconomic indicators. Therefore, my statistical approach involved two steps. First, to show the level of a woman's LTR of dying, the dataset on maternal mortality was disaggregated into the commonly used four policy‐relevant socioeconomic factors: such as wealth, education, regional states and residence. Second, to model disparities, the rate ratio (*R*) was used [29]. Household wealth was also examined using the constructed wealth (asset) indexes provided in all EDHS, comprising five dimensions of socioeconomic positions (i.e. from lowest to highest wealth quintiles) [2].

As per the recommendation of estimation of maternal mortality in the case of the sisterhood method [30], maternal mortality was determined based on the following calculations:

- LTR = 1 − (1 − MMR)^TFR^; or LTR = *r*/*B*);
  where MMR is the maternal mortality ratio, TFRis the total fertility rate, *r* is the number of pregnancy‐related deaths, and *B* is the sister unit of exposure.
- In addition, the followings calculations were done:
  - Probability of avoiding maternal death (*p*), that is *p* = 1 − LTR; or MMR = 1 − *p* ^1/TFR^;
  - Sister unit of exposure (*B*), *B* = *r*/LTR;
  - Standard error of LTR (SE), SE (LTR) = √(*r*/*B*) (1 − *r*/*B*)/*B*;
  - 95% CI of LTR, that is 95% CI of LTR = ±1.96 SE (LTR);
  - 95% CI of MMR for both the lower and upper limits, that is 95% CI of MMR = LTR/TFR (Supporting Information [Link], [Link], [Link], [Link]).

The 95% confidence interval (CI) was also used to determine statistical significance between the two consecutive years. If the CI did not overlap, there were a woman's LTR disparities in maternal mortality, and there is a statistically significant difference between the two consecutive years. However, if the CI overlaps, there were no woman's LTR disparities in maternal mortality, and there is no statistically significant difference between the two consecutive years.

To assess a woman's LTR disparities in maternal mortality in Ethiopia, rate ratio (*R*) was used as per the WHO's Health Equity Assessment Toolkit plus software technical notes and software (HEAT plus) [27], and the WHO's handbook on health inequality monitoring with a special focus on low‐ and middle‐income countries [31].

### Ethical considerations

Ethical clearance for EDHS was obtained by the Ethiopian Health and Nutrition Research Institute (EHNRI) Review Board, the National Research Ethics Review Committee (NRERC) at the Ministry of Science and Technology, the Institutional Review Board (IRB) of ICF International, Inc. and the United States Centres for Disease Control and Prevention (CDC). Therefore, permission to use the publicly available EDHS datasets was obtained and approved by ICF International, Inc. DHS dataset access authorization letter on Nov 03, 2021 (AuthLetter_161444).

## RESULTS

### Socio‐demographic characteristics of respondents across the survey years in Ethiopia

From 61,610 respondents, 795 deaths were reported. The study participants (*n* = 61,610) were from the nine sub‐national regional states and two city council administrations. The age group 15–19‐year old represents about a quarter of the respondents (21.6%–24.1%). Table 1 presents the socio‐demographic characteristics of respondents across the survey years from 2000 to 2016 in Ethiopia.

**TABLE 1:** Socio‐demographic characteristics of respondents across the survey years from 2000 to 2016 in Ethiopia

		Survey years							
		2000 (*n* = 15,367)		2005 (*n* = 14,070)		2011 (*n* = 16,490)		2016 (*n* = 15,683)	
Socio‐demographic variables	Category	Freq.	%	Freq.	%	Freq.	%	Freq.	%
Age of women (years)	15–19	3710	24.1	3266	23.2	4009	24.3	3381	21.6
	20–24	2860	18.6	2547	18.1	2931	17.7	2762	17.6
	25–29	2585	16.8	2517	17.9	3147	19.1	2957	18.9
	30–34	1841	12.0	1808	12.8	2054	12.4	2345	15.0
	35–39	1716	11.2	1602	11.4	1916	11.6	1932	12.3
	40–44	1392	9.1	1187	8.4	1261	7.6	1290	8.2
	45–49	1264	8.2	1143	8.1	1196	7.2	1017	6.5
Place of residence	Urban	2791	18.2	2499	17.8	3947	23.9	3476	22.2
	Rural	12,576	81.8	11,571	82.2	12,568	76.1	12,207	77.8
Educational status	No education	11,551	75.2	9271	65.9	8394	50.8	7498	47.8
	Primary	2425	15.8	3123	22.2	6276	38.0	5490	35.0
	Secondary	1304	8.5	1481	10.5	1117	6.8	1817	11.6
	Higher	87	0.6	194	1.4	728	4.4	877	5.6
Wealth quintile	Lowest	2825	18.4	2428	17.3	2986	18.1	2633	16.8
	Second	2881	18.7	2643	18.8	3041	18.4	2809	17.9
	Middle	2957	19.2	2732	19.4	3031	18.4	2978	19.0
	Fourth	3013	19.6	2647	18.8	3215	19.5	3100	19.8
	Highest	3675	23.9	3621	25.7	4242	25.7	4163	26.5
Regional states	Tigray	970	6.3	919	6.5	1104	6.7	1129	7.2
	Afar	178	1.2	146	1.0	145	0.9	128	0.8
	Amhara	3820	24.9	3482	24.7	4433	26.8	3714	23.7
	Oromiya	5936	38.6	5010	35.6	6011	36.4	5701	36.4
	Somali	175	1.1	486	3.5	329	2.0	459	2.9
	Benishangul‐Gumuz	159	1.0	124	0.9	174	1.1	160	1.0
	SNNPR	3284	21.4	2995	21.3	3236	19.6	3288	21.0
	Gambella	39	0.3	44	0.3	69	0.4	44	0.3
	Harari	41	0.3	39	0.3	49	0.3	38	0.2
	Addis Ababa	684	4.5	756	5.4	896	5.4	930	5.9
	Dire Dawa	80	0.5	69	0.5	69	0.4	90	0.6

*Note*: Freq. = weighted frequency; *n* = number.

### A woman's lifetime risks (chance) of dying from maternal causes in Ethiopia

#### Residence‐based woman's lifetime risks of dying

In 2000, the highest woman's LTR of dying from maternal causes over the woman's lifespan or reproductive years was observed among women in rural areas (1 in 32 women). Similarly, in 2016, it was also 1 in 111 women in rural areas. However, there were no statistically significant differences between the rural and urban areas (Table 2) (Supporting Information [Link], [Link], [Link], [Link]).

**TABLE 2:** A woman's lifetime risk (LTR) of dying from maternal causes from 2000 to 2016 in Ethiopia

	Survey years																			
	2000					2005					2011					2016				
Dimensions of disparity	No of death	No of sisters live	LTR	LTR 1 in:	MMR per 100,000 live births (95% CI)	No of death	No of sister alive	LTR	LTR 1 in:	MMR per 100,000 live births (95% CI)	No of death	No of sister alive	LTR	LTR 1 in:	MMR per 100,000 live births (95% CI)	No of death	No of sister alive	LTR	LTR 1 in:	MMR per 100,000 live births (95% CI)
**Place of residence**																				
Rural	206	17,079	0.031	1:32	581 (500, 648)	186	19,932	0.024	1:42	449 (383, 516)	170	23,231	0.019	1:53	399 (337, 455)	82	26,674	0.009	1:111	196 (154, 237)
Urban	48	8761	0.016	1:63	298 (204, 370)	14	4566	0.009	1:111	167 (80, 254)	43	8014	0.015	1:67	315 (219, 406)	18	6702	0.007	1:143	153 (80, 224)
**Educational status**																				
No education	207	16,178	0.030	1:33	563 (482, 630)	149	14,775	0.023	1:44	430 (357, 495)	122	14,643	0.019	1:53	399 (327, 465)	65	13,320	0.011	1:91	240 (185, 296)
Primary	30	5412	0.019	1:53	355 (222, 482)	39	7686	0.019	1:53	355 (243, 461)	86	15,625	0.020	1:50	420 (327, 507)	30	12,950	0.008	1:125	175 (111, 237)
Secondary	13	5154	0.009	1:111	167 (78, 259)	7	3231	0.008	1:125	149 (43, 253)	5	3260	0.006	1:167	126 (19, 231)	17	5158	0.012	1:83	262 (137, 385)
Higher	1	252	0.008	1:125	149 (−148, 444)	1	404	0.007	1:143	130 (114, 373)	1	1745	0.002	1:500	42 (−35, 120)	1	1915	0.002	1:500	44 (−28, 115)
**Wealth quintile**																				
Lowest	29	3396	0.020	1:50	373 (236, 505)	33	3813	0.021	1:48	392 (258, 464)	46	5374	0.021	1:48	441 (311, 564)	22	4413	0.012	1:83	262 (35, 372)
Second	56	3628	0.038	1:26	715 (522, 885)	44	4495	0.024	1:42	449 (314, 575)	48	5424	0.023	1:44	484 (345, 614)	15	4662	0.008	1:125	175 (85, 263)
Middle	48	4585	0.027	1:37	506 (359, 641)	41	4787	0.022	1:46	411 (281, 535)	29	5639	0.013	1:77	272 (172, 370)	29	5650	0.013	1:77	284 (181, 385)
Fourth	67	4285	0.041	1:24	772 (582, 937)	40	4653	0.022	1;46	411 (284, 531)	38	6393	0.016	1:63	336 (227, 440)	22	6223	0.009	1:111	196 (115, 276)
Highest	52	10,001	0.015	1:67	280 (204, 350)	38	6780	0.016	1:63	298 (202, 391)	48	8608	0.016	1:63	336 (240, 427)	24	8156	0.008	1:125	195 (107, 241)
**Regional states**																				
Tigray	17	2020	0.021	1:48	392 (207, 571)	13	1506	0.023	1:44	430 (201, 651)	17	1955	0.023	1:44	484 (254, 705)	9	2159	0.011	1:91	240 (85, 394)
Afar	18	915	0.046	1:22	868 (467, 1237)	1	208	0.012	1:83	223 (−207, 650)	0	225	0	0	0	0	174	0	0	0
Amhara	35	2032	0.029	1:35	544 (361, 713)	54	5569	0.025	1:40	468 (340, 587)	42	7966	0.014	1:72	293 (204, 379)	18	6805	0.007	1:143	153 (85, 222)
Oromiya	50	4807	0.028	1:36	525 (376, 661)	72	9231	0.021	1:48	392 (302, 476)	82	11,763	0.019	1:53	399 (311, 482)	58	11,059	0.014	1:71	306 (228, 380)
Somali	17	1503	0.029	1:35	544 (287, 787)	7	820	0.020	1:50	374 (98, 643)	4	610	0.017	1:59	357 (11, 697)	2	959	0.006	1:167	131 (−40, 300)
Benishangul‐Gumuz	23	1442	0.042	1:24	791 (469, 1087)	4	187	0.056	1:18	1062 (54, 2021)	0	284	0	0	0	0	291	0	0	0
SNNPR	31	3396	0.023	1:44	430 (208, 572)	46	5313	0.022	1:46	411 (288, 527)	46	6309	0.019	1:53	399 (284, 508)	21	6315	0.009	1:111	196 (115, 276)
Gambella	18	1075	0.043	1:23	811 (437, 1155)	0	56	0	0	0	0	110	0	0	0	0	56	0	0	0
Harari	12	1642	0.019	1:53	355 (152, 552)	0	73	0	0	0	0	88	0	0	0	0	66	0	0	0
Addis Ababa	16	3877	0.012	1:83	223 (113, 332)	4	1481	0.008	1:125	149 (7, 290)	9	1730	0.015	1:67	315 (113, 512)	3	1687	0.005	1:200	109 (−11, 228)
Dire Dawa	14	1894	0.019	1:53	355 (168, 535)	0	120	0	0	0	0	115	0	0	0	0	156	0	0	0
Ethiopia	263	32,609	0.046	1:22	871 (703, 1039)	197	38,392	0.039	1:26	673 (548, 799)	217	34,984	0.036	1:28	676 (541, 810)	118	33,272	0.019	1:52	412 (273, 551)

*Note*: MMR = age‐adjusted maternal mortality ratio per 100,000 live births; 95% CI = 95% lower and upper limits of the estimate; LTR = lifetime risk of maternal mortality.

#### Regional states‐based woman's lifetime risks of dying

In 2000, the highest woman's risk of dying from maternal causes was seen in the Afar regional state (1 in 22 women); but, in 2016, it was 1 in 71 women in Oromiya regional state. However, there were no statistically significant differences among the other regional states (Table 2) (Supporting Information [Link], [Link], [Link], [Link]).

#### Educational status‐based woman's lifetime risks of dying

In 2000, the highest woman's LTR of dying from maternal causes was detected among women with lower educational status (1 in 33 women); but, in 2016, it was 1 in 82 women with secondary education. However, there were no statistically significant differences among the other three sub‐categories, except among those with higher education (Table 2) (Supporting Information [Link], [Link], [Link], [Link]).

#### Wealth‐based woman's lifetime risks of dying

In 2000, the highest woman's risk of dying from maternal causes was identified among richer women (1 in 24 women); but, in 2016, it was 1 in 77 women with middle income. However, there were no statistically significant differences (Table 2) (Supporting Information [Link], [Link], [Link], [Link]).

### A change in lifetime risk of dying from maternal causes in Ethiopia

At the national level, between the earlier (2000) and latest (2016) survey years, a woman's LTR of dying from maternal causes declined by 59%. That means the change in a woman's LTR of dying from maternal causes nearly halved from 4.6% (1 in 22 women) to 1.9% (1 in 52 women), respectively (Table 3) (Supporting Information [Link], [Link], [Link], [Link]).

**TABLE 3:** A change in lifetime risk (LTR) of dying from maternal causes and maternal mortality ratio (MMR) in 2000 and 2016, Ethiopia

		Survey years					
		LTR in earlier vs. latest surveys			MMR in earlier vs. latest surveys		
Dimensions of disparity	Category	2000	2016	Change	2000	2016	Change
Place of residence	Urban	0.031	0.009	0.022 (71%)	581	196	385 (66%)
	Rural	0.016	0.007	0.009 (56%)	298	153	145 (49%)
Educational status	No education	0.03	0.011	0.019 (63%)	563	240	323 (58%)
	Primary	0.019	0.008	0.011 (58%)	355	175	180 (51%)
	Secondary	0.009	0.012	−0.003 (33%)	167	262	−95 (57%)
	Higher	0.008	0.002	0.006 (75%)	149	44	105 (70%)
Wealth quintile	Lowest	0.02	0.012	0.008 (40%)	373	262	111 (29%)
	Second	0.038	0.008	0.030 (79%)	715	175	540 (76%)
	Middle	0.027	0.013	0.014 (52%)	506	284	252 (50%)
	Fourth	0.041	0.009	0.032 (78%)	772	196	576 (75%)
	Highest	0.015	0.008	0.007 (47%)	280	195	85 (31%)
Regional states	Tigray	0.021	0.011	0.010 (48%)	392	240	152 (39%)
	Afar	0.046	0	(100%)	868	0	(100%)
	Amhara	0.029	0.007	0.022 (76%)	544	153	391 (72%)
	Oromiya	0.028	0.014	0.014 (50%)	525	306	219 (42%)
	Somali	0.029	0.006	0.023 (79%)	544	131	413 (76%)
	Benishangul‐Gumuz	0.042	0	(100%)	791	0	(100%)
	SNNPR	0.023	0.009	0.014 (61%)	430	196	234 (55%)
	Gambella	0.043	0	(100%)	811	0	(100%)
	Harari	0.019	0	(100%)	355	0	(100%)
	Addis Ababa	0.012	0.005	0.007 (58%)	223	109	114 (51%)
	Dire Dawa	0.019	0	(100%)	355	0	(100%)
National		0.046	0.019	0.027 (59%)	871	412	459 (53%)

*Note*: LTR = lifetime risk of maternal mortality; MMR = maternal mortality ratio per 100,000 live births.

#### Residence‐driven change in lifetime risk

There was a consecutive decline in a woman's LTR of dying in terms of the place of residence. For example, from 2000 to 2016, in rural areas, a woman's LTR of dying declined from 1 in 32 women to 1 in 111 women. In urban areas, it also declined from 1 in 63 women to 1 in 143 women (Table 3) (Supporting Information [Link], [Link], [Link], [Link]).

#### Regional states‐driven change in lifetime risk

There was also a consecutive decline in a woman's LTR of dying among geographical areas (regional states). Yet, the difference was wide. For example, in a recent survey in 2016, a woman's LTR of dying in the Oromiya regional state was 1.4% (1 in 71 women), although there was a decline of 50%, from 2.8% (1 in 36 women) in 2000, (Table 3) (Supporting Information [Link], [Link], [Link], [Link]).

#### Education‐driven change in lifetime risk

From 2000 to 2016, a woman's LTR of dying among women with lower educational status declined from 1 in 33 women to 1 in 91 women. In addition, among those with higher education, it declined from 1 in 125 women to 1 in 500 women. However, over the past 20 years, a woman's LTR of dying did not decline among those with secondary education; rather, it increased from 1 in 111 women to 1 in 83 women (Table 3) (Supporting Information [Link], [Link], [Link], [Link]).

#### Wealth‐driven change in lifetime risk

From 2000 to 2016, the wealth‐driven woman's LTR of dying among the poorest women declined from 1 in 50 women to 1 in 83 women. Among the richest women also, a woman's LTR of dying declined from 1 in 125 women to 1 in 500 women (Table 3) (Supporting Information [Link], [Link], [Link], [Link]).

### A risk estimate for woman's lifetime risk disparities in maternal mortality in Ethiopia

#### Residence‐related risk estimate

Urban–rural‐related woman's LTR disparities in maternal mortality in Ethiopia decreased by about 35%, from 2% in 2000 to 1.3% in 2016. However, in 2005, it was 3‐times higher among women in rural areas (*R* = 2.7 [95% CI: 2.3, 3.1]). In 2016 also, woman's LTR disparities in maternal mortality were 1.3‐times higher among women in rural areas (*R* = 1.3 [95% CI: 1.1, 1.6]) (Table 4).

**TABLE 4:** A risk estimate for woman's lifetime risk disparities in maternal mortality from 2000 to 2016 in Ethiopia

	Survey years			
Dimensions of disparity	2000 Risk estimate (95% CI)	2005 Risk estimate (95% CI)	2011 Risk estimate (95% CI)	2016 Risk estimate (95% CI)
Place of residence	2.0 (1.7, 2.2)	2.7 (2.3, 3.1)	1.3 (1.1, 1.5)	1.3 (1.1, 1.6)
Educational status	3.8 (3.3, 4.3)	3.3 (2.8, 3.8)	9.5 (8.2, 10.8)	5.5 (4.5, 6.5)
Wealth quintile	1.3 (1.2, 1.5)	1.3 (1.1, 1.5)	1.3 (1.1, 1.5)	1.3 (1.1, 1.6)
Regional states	3.9 (3.4, 4.4)	7.1 (6.15, 8.1)	1.7 (1.4, 1.9)	1.6 (1.3, 1.9)

*Note*: Risk estimate = the point estimate for the rate ratio; 95% CI = the 95% lower and upper bound for the point estimate of the rate ratio.

#### Regional states‐related risk estimate

Regional states‐related woman's LTR disparities in maternal mortality declined by 59%, from 3.9% in 2000 to 1.6% in 2016. However, in 2000, it was 4‐times higher among women in Afar regional state (*R* = 3.9 [95% CI: 3.48, 4.4]). Similarly, in 2005, it was 7‐times higher among women in Benishangul‐Gumuz regional state (*R* = 7.1 [95% CI: 6.15, 8.1]). Moreover, in 2016, woman's LTR disparities in maternal mortality were 2‐times higher among women in Oromiya regional state (*R* = 1.6 [95% CI: 1.3, 1.9]) (Table 4).

#### Education‐related risk estimate

Education‐related woman's LTR disparities in maternal mortality did not reduce, rather, elevated by 42%, from 3.8% in 2000 to 5.5% in 2016. Moreover, in 2011, woman's LTR disparities in maternal mortality were 10‐times higher among women with lower educational status (*R* = 9.5 [95% CI: 8.2, 10.8]). In 2016 also, it was 6‐times higher among women with lower educational status (*R* = 5.5 [95% CI: 4.5, 6.5]) (Table 4).

#### Wealth‐related risk estimate

Wealth‐related a woman's LTR disparities in maternal mortality fell‐off by 35%, from 2% in 2000 to 1.3% in 2016. However, in 2005, a woman's LTR disparities in maternal mortality were 3‐times higher among the poorest women (*R* = 2.7 [95% CI: 2.3, 3.1]). On the other side, from 2011 up to 2016, the burden of a woman's LTR disparities in maternal mortality among the poorest women remained the same or unchanged (*R* = 1.3 [95% CI: 1.2, 1.5]); and (*R* = 1.3 [95% CI: 1.1, 1.6]), respectively (Table 4).

## DISCUSSION

In summary, using the four rounds of EDHS, this study intended to assess a woman's LTR disparities in maternal mortality in Ethiopia. Nearly, 795 maternal deaths were identified over the past 20 years, from 2000 to 2016. However, a woman's LTR of dying declined by 59%. The change in a woman's LTR of dying over time could be by the fact that the improvement of most maternal health‐care indicators as a result of the health reforms in Ethiopia [20]. However, a woman's LTR disparities in maternal mortality have been substantial. Indeed, indicating that women from rural and poorest areas and even women with lower educational status are still at increased a woman's LTR of dying from maternal causes.

The findings of a woman's LTR disparities in maternal mortality in the present study were different from the finding from Bonke district in rural south‐west Ethiopia, where the level of woman's LTR disparities in maternal mortality was found to be too high, that is 10% (1 in 10 women) [32]. This difference could be because the population of Bonke district resided in isolated and remote rural villages where no access to basic and comprehensive emergency obstetric care, there was limited transportation, and high household poverty. Therefore, the findings of the current study highlight new insight into using the evidence of a woman's LTR disparities in maternal mortality in Ethiopia. In addition, a woman's LTR disparities in maternal mortality, as used in this approach, should be taken into account by policymakers to identify vulnerable groups considering how to set up the priority for the country with limited‐resource as the MMR or MM rate was not constant throughout all levels [4].

Even though the observed woman's LTR disparities in maternal mortality across the survey years (2000–2016) appear to decline in Ethiopia, in the present study, the overall woman's LTR disparities in maternal mortality were considerably high in 2016. Yet, this finding was inconsistent with a study conducted in Kersa, Eastern Ethiopia, in which a woman's LTR disparities in maternal mortality were higher by half than the national figure (1 in 23 women vs. 1 in 52 women). The possible reason for the higher woman's LTR disparities in maternal mortality in the Kersa district was that the data were longitudinal data, but the data for EDHS were cross‐sectional data. That means each household in Kersa district Demographic Surveillance and Survey site (HDSS) followed regularly and all reproductive‐aged women who died could be recorded. There could also be no missing maternal death data. In addition, the population residing in the Kersa surveillance site most likely have better knowledge of the utilization of available maternal health‐care services, which in turn could have a positive influence on maternal health and could lead them to report maternal death if occurs [7]. Nonetheless, the finding of a woman's LTR of dying from maternal causes in Ethiopia was lower than the average in sub‐Saharan Africa and slightly lower than the average in Africa, but, it was higher than the global average [3]. Therefore, although there is an encouraging move in reducing a woman's LTR disparities in maternal mortality, still, it requires more government action.

From the educational status perspective, contrary to the hypothesized association that a woman's LTR of dying was more common among women with lower educational status; the findings indicate that there was an increment in a woman's LTR of dying among women with secondary education. Therefore, this might be explained that due to more accurate reporting of maternal mortality by women with higher education than women with lower educational status. Nevertheless, this was not expected as educated and urban women are more likely to have good infrastructure, have paid work and have higher economic development than uneducated women. However, a similar study from Kenya on maternal mortality evidenced that more maternal mortality was reported by educated and urban women than among uneducated and rural residents [29]. Therefore, as there could be under‐reporting, the findings should be interpreted with care. However, the possible confounding factors for an increase in a woman's LTR of dying among secondary school girls could be young adolescent girls who may face school dropouts and then early marriages by age 18. As a result, the rates of adolescent pregnancies remain high among girls in low‐ and middle‐income countries [33].

From the geographical (regional states) perspective, in a recent survey in 2016, even though the women who were living in Addis Ababa (the capital city of Ethiopia) were with the smallest a woman's LTR disparities in maternal mortality compared with other regions in Ethiopia; this finding has highly differed from the report in northern Europe and northern America that showed a lower woman's LTR disparities in maternal mortality, that is 1 in 4800 women and even in Australia and New Zealand, it was 1 in 7800 women [3]. This could be explained by the fact that developed countries could have reduced a woman's LTR disparities in maternal mortality due to the overall level of development, developed health‐care infrastructure, low levels of the fertility rate, and the women could be more exposed to mass media which provides more information on modern methods of family planning and utilization of maternal health‐care services. Therefore, as siblings and their dead sister might not be living in the same place, findings on residence could be lower or higher reporting and should also be interpreted with care [34].

From the residence perspective, rural women in Ethiopia, with poor access to healthcare were often more likely to experience a higher incidence of a woman's LTR disparities in maternal mortality than their urban counterparts. However, this was expected in rural areas and could be explained that women in rural areas are more likely to have poor health‐care infrastructure, a high number of uneducated women, and lower socioeconomic development than urban women. Therefore, these findings are in agreement with other studies done in south Ethiopia, which demonstrated that household poverty and geographical location could be the reasons for high woman's LTR disparities in maternal mortality [32].

From the wealth status perspective, the present findings were consistent with reports that indicated a woman's LTR disparities in maternal mortality were more common among mistreated, neglected and poor women in developing countries than women who lived in developed nations. However, in 2019, in the United States of America, findings demonstrated that mistreatment of women across the continuum of maternal care which may be manifested as neglect, lack of consent, and/or coercion, and lack of access to comprehensive postpartum support, and contributing to the increase from 20 to 24 maternal deaths per 100,000 live births [28, 35–37]. Therefore, the Ethiopian Ministry of Health should learn and strive towards addressing the underlying factors (i.e. obstetric discrimination towards the poor) and reducing a woman's LTR disparities in maternal mortality.

### Strengths and limitations

A nationally representative population‐based survey dataset was used to show the marked woman's LTR disparities in maternal mortality across the country, including in regional states, thereby the findings could be generalizable to the women in Ethiopia. This study also provides a big picture of a woman's LTR disparities in maternal mortality that should be interpreted in light of a woman's LTR of maternal death per woman rather than the ratio with live births (i.e. MMR or MM rate) [6], by taking into account both the probability of becoming pregnant and the probability of dying as a result of that pregnancy [38]. This is because, unless a statistical test, like rate ratio (*R*), is used, other reporting could be misleading and less informative for decision‐making [39]. However, this study has some limitations. Some of the estimates relying on small numbers of maternal mortality may be unreliable. The analysis was also limited only to variables within the dataset. In addition, due to the rare nature of maternal mortality and the wider CI for the estimates [39], the findings should be interpreted with care [39]. However, there are strong reasons that the findings of this study could be more informative and insightful, as is evidenced by similar studies and earlier procedures, indicating the accuracy and appropriateness of the rate ratio (*R*) [4].

### Policy implications

In this study, the measurement of a woman's LTR showed the number of maternal mortalities that could potentially be averted. In addition, a woman's LTR disparities in maternal mortality remain a maternal health equity issue. However, the application of a woman's LTR of dying is inappropriately represented as not the MM rate or MMR. This study has taken an in‐depth view into maternal mortality as considerations of a woman's LTR of dying have gone beyond just the rate and ratio of maternal mortality often used. As similar studies indicated that without further efforts in the rural and poorest areas, where the majority of maternal mortality is reported, Ethiopia's effort to achieve the 2030 SDGs could be hindered [20]. In addition, as evidenced by other studies in Kenya, threatening factors, like the current COVID‐19 pandemic [40], and some unrest and conflicts in some parts of the country could worsen and disrupt the appropriate delivery of maternal health‐care services and sustain a woman's LTR disparities in maternal mortality [41].

### Implications for future research

This study raises several opportunities for future research in terms of concept validation. More research will be necessary to refine and further elaborate the findings of this study. First, this study has implications for further research on the drivers as well as effective actions to curb a woman's LTR disparities in maternal mortality. Second, this study has also implications for future research and could also be a possible area of future research and debate in that using a woman's LTR of dying is more precise and informative than the MMR and/or MM rate. Therefore, the study could also be extended longitudinally and comparatively as a policy input in the application of a woman's LTR of dying versus MMR and/or MM rate.

## CONCLUSIONS

This study evidenced that even though a woman's LTR (chance) of dying due to maternal causes over a woman's life course or reproductive years reduced from 2000 to 2016, Ethiopian women experienced high LTR disparities in maternal mortality which varies across socioeconomic groups. A woman's chance of dying due to maternal causes is closely connected to her social and economic status and the geographic area in which the woman lives. For example, the socioeconomic‐related factors that drive a woman's LTR disparities in maternal mortality were concentrated among women with lower educational status and among poor women, and in rural areas. Therefore, equity‐oriented maternal healthcare is recommended to reduce the effects of structural inequities, such as poverty, and the inequitable distribution of the socioeconomic factors of maternal health, such as income, education, residence and regional states, that sustain the existing a woman's LTR disparities in maternal mortality.

## AUTHOR CONTRIBUTIONS

Moges Tadesse Borde conceived and developed the study idea and design, designed the methodology, performed a literature review and participated in data extraction, data curation and formal analysis, wrote the initial draft manuscript and wrote the review and editing of the final manuscript, revised and approved the final draft for publication.

## CONFLICT OF INTEREST

I declare that I have no competing interests.

## ETHICS STATEMENT

Ethical clearance for Ethiopian Demographic and Health Survey (EDHS) was obtained by the Ethiopian Health and Nutrition Research Institute (EHNRI) Review Board, the National Research Ethics Review Committee (NRERC) at the Ministry of Science and Technology, the Institutional Review Board (IRB) of ICF International, Inc. and the United States Centres for Disease Control and Prevention (CDC). Therefore, permission to use the publicly available EDHS datasets was obtained and approved by ICF International, Inc. DHS Dataset access authorization letter on NOV 03, 2021 (AuthLetter_161444).

## Supporting information

Supplementary file 1 Estimation of lifetime risks of maternal mortality for the year 2000, Ethiopia

Supplementary file 2 Estimation of lifetime risks of maternal mortality for the year 2005, Ethiopia

Supplementary file 3 Estimation of lifetime risks of maternal mortality for the year 2011, Ethiopia

Supplementary file 4 Estimation of lifetime risks of maternal mortality for the year 2016, Ethiopia

## Data Availability

Data are available in a public, open‐access repository. The dataset is freely available at: https://dhsprogram.com/data/available‐datasets.cfm. All other relevant data are available within the paper.
