# Supplementary material for: A woman's lifetime risk disparities in maternal mortality in Ethiopia
Source: Public Health Chall. 2023 Jan 13;2(1):e56. doi: 10.1002/puh2.56 (PMC12039551; doi:10.1002/puh2.56)
Supplement: Supplementary file 1 — Estimation of lifetime risks of maternal mortality for the year 2000, Ethiopia [file PUH2-2-e56-s001.docx]

**Supplementary file 1.** Estimation of lifetime risks of maternal mortality for the year 2000, Ethiopia

**Place of residence**

**Urban**

| **Age of respondents**  **in years** | **No of respondents** | **No. of sisters survived**  **(>15 years)** | **No. of sisters died from all maternal causes** | **No. of pregnancy-related deaths** | **Adjustment**  **factors** | **Sister unit of exposure (E)** |
| --- | --- | --- | --- | --- | --- | --- |
|  |  | **(A)** | **(B)** | **(C)** | **(D)** | **(E=A*D)** |
| 15-19 | 816 | 2562* | 47 | 8 | 0.107 | 274 |
| 20-24 | 542 | 1702* | 77 | 13 | 0.206 | 351 |
| 25-29 | 510 | 1551 | 69 | 7 | 0.343 | 532 |
| 30-34 | 290 | 1226 | 52 | 10 | 0.503 | 617 |
| 35-39 | 288 | 871 | 34 | 6 | 0.664 | 578 |
| 40-44 | 189 | 557 | 30 | 1 | 0.802 | 447 |
| 45-49 | 156 | 292 | 18 | 3 | 0.900 | 263 |
| **Total** | **2791** | **8761** | **327** | **48** |  | **3061** |

* Adjusted number of sisters by multiplying the average number of sisters for respondents aged 25–49 (i.e., 3.14) by the number of respondents (age group 15–19 and 20–24). Originally the number of sisters was 1409 for the age 15–19 years, and 1742 for 20–24 years. LTR = 48/3061 = 0.016, TFR = 5.4 for 7-previous years, and MM Ratio = 1-(1- LTR)^1/TFR^ = 298/100,000 LB (95%CI: 204, 370).

**Rural**

| **Age of respondents**  **in years** | **No of respondents** | **No. of sisters survived (>15 years)** | **No. of sisters died from all maternal causes** | **No. of pregnancy-related deaths** | **Adjustment**  **factors** | **Sister unit of exposure (E)** |
| --- | --- | --- | --- | --- | --- | --- |
|  |  | **(A)** | **(B)** | **(C)** | **(D)** | **(E=A*D)** |
| 15-19 | 2894 | 3936* | 117 | 26 | 0.107 | 421 |
| 20-24 | 2317 | 3151* | 127 | 44 | 0.206 | 649 |
| 25-29 | 2075 | 2945 | 141 | 54 | 0.343 | 1010 |
| 30-34 | 1551 | 2549 | 126 | 43 | 0.503 | 1282 |
| 35-39 | 1428 | 2081 | 92 | 22 | 0.664 | 1382 |
| 40-44 | 1203 | 1477 | 62 | 14 | 0.802 | 1185 |
| 45-49 | 1108 | 940 | 46 | 3 | 0.900 | 846 |
| **Total** | **12576** | **17079** | **711** | **206** |  | **6775** |

* Adjusted number of sisters by multiplying the average number of sisters for respondents aged 25–49 (i.e., 1.36) by the number of respondents (age group 15–19 and 20–24). Originally the number of sisters was 2567 for the age 15–19 years, and 3222 for 20–24 years. LTR = 206/6775 = 0.031, TFR = 5.4 for 7-previous years, and MM Ratio = 1-(1- LTR)^1/TFR^ = 581/100,000 LB (95%CI: 500, 648).

**Educational status**

**No education**

| **Age of respondents**  **in years** | **No of respondents** | **No. of sisters survived (>15 years)** | **No. of sisters died from all maternal causes** | **No. of pregnancy-related deaths** | **Adjustment**  **factors** | **Sister unit of exposure (E)** |
| --- | --- | --- | --- | --- | --- | --- |
|  |  | **(A)** | **(B)** | **(C)** | **(D)** | **(E=A*D)** |
| 15-19 | 2265 | 3171* | 114 | 27 | 0.107 | 339 |
| 20-24 | 2015 | 2821* | 134 | 43 | 0.206 | 581 |
| 25-29 | 1812 | 2832 | 140 | 49 | 0.343 | 971 |
| 30-34 | 1459 | 2535 | 130 | 45 | 0.503 | 1275 |
| 35-39 | 1484 | 2183 | 106 | 27 | 0.664 | 1450 |
| 40-44 | 1298 | 1596 | 67 | 10 | 0.802 | 1280 |
| 45-49 | 1218 | 1040 | 54 | 6 | 0.900 | 936 |
| **Total** | 11551 | 16178 | **745** | **207** |  | 6832 |

* Adjusted number of sisters by multiplying the average number of sisters for respondents aged 25–49 (i.e., 1.4) by the number of respondents (age group 15–19 and 20–24). Originally the number of sisters was 2334 for the age 15–19 years, and 2946 for 20–24 years. LTR = 207/6832 = 0.030, TFR = 5.4 for 7-previous years, and MM Ratio = 1-(1- LTR)^1/TFR^ = 563/100,000 LB (95%CI: 482, 630).

**Primary**

| **Age of respondents**  **in years** | **No of respondents** | **No. of sisters survived (>15 years)** | **No. of sisters died from all maternal causes** | **No. of pregnancy-related deaths** | **Adjustment**  **factors** | **Sister unit of exposure (E)** |
| --- | --- | --- | --- | --- | --- | --- |
|  |  | **(A)** | **(B)** | **(C)** | **(D)** | **(E=A*D)** |
| 15-19 | 977 | 2179* | 29 | 3 | 0.107 | 233 |
| 20-24 | 495 | 1104* | 38 | 10 | 0.206 | 227 |
| 25-29 | 443 | 780 | 25 | 8 | 0.343 | 268 |
| 30-34 | 258 | 593 | 21 | 4 | 0.503 | 298 |
| 35-39 | 155 | 392 | 11 | 2 | 0.664 | 260 |
| 40-44 | 62 | 247 | 14 | 3 | 0.802 | 198 |
| 45-49 | 35 | 117 | 5 | 0 | 0.900 | 105 |
| **Total** | 2425 | 5412 | **143** | **30** |  | 1590 |

* Adjusted number of sisters by multiplying the average number of sisters for respondents aged 25–49 (i.e., 2.23) by the number of respondents (age group 15–19 and 20–24). Originally the number of sisters was 811 for the age 15–19 years, and 934 for 20–24 years.

LTR = 30/1590 = 0.019, TFR = 5.4 for 7-prevous years, and MM Ratio = 1-(1- LTR)^1/TFR^ = 355/100,000 LB (95%CI: 222, 482).

**Secondary**

| **Age of respondents**  **in years** | **No of respondents** | **No. of sisters survived (>15 years)** | **No. of sisters died from all maternal causes** | **No. of pregnancy-related deaths** | **Adjustment**  **Factors** | **Sister unit of exposure (E)** |
| --- | --- | --- | --- | --- | --- | --- |
|  |  | **(A)** | **(B)** | **(C)** | **(D)** | **(E=A*D)** |
| 15-19 | 468 | 1849* | 21 | 3 | 0.107 | 198 |
| 20-24 | 336 | 1327* | 41 | 4 | 0.206 | 273 |
| 25-29 | 299 | 816 | 45 | 3 | 0.343 | 280 |
| 30-34 | 105 | 588 | 21 | 3 | 0.503 | 296 |
| 35-39 | 64 | 336 | 10 | 0 | 0.664 | 223 |
| 40-44 | 24 | 176 | 11 | 0 | 0.802 | 141 |
| 45-49 | 9 | 62 | 4 | 0 | 0.900 | 56 |
| **Total** | 1305 | 5154 | **153** | **13** |  | 1467 |

* Adjusted number of sisters obtained by multiplying the average number of sisters for respondents aged 25–49 (i.e., 3.95) by the number of respondents (age group 15–19 and 20–24). Originally the number of sisters was 821 for the age 15–19 years, and 1021 for 20–24 years. LTR = 13/1467 = 0.009, TFR = 5.4 for 7-previous years, and MM Ratio = 1-(1- LTR)^1/TFR^ = 167/100,000 LB (95%CI: 78, 259).

**Higher**

| **Age of respondents**  **in years** | **No of respondents** | **No. of sisters survived (>15 years)** | **No. of sisters died from all maternal causes** | **No. of pregnancy-related deaths** | **Adjustment**  **factors** | **Sister unit of exposure (E)** |
| --- | --- | --- | --- | --- | --- | --- |
|  |  | **(A)** | **(B)** | **(C)** | **(D)** | **(E=A*D)** |
| 15-19 | 0 | 0 | 0 | 0 | 0.107 | 0 |
| 20-24 | 14 | 23 | 1 | 0 | 0.206 | 5 |
| 25-29 | 32 | 80 | 1 | 1 | 0.343 | 27 |
| 30-34 | 18 | 67 | 3 | 0 | 0.503 | 34 |
| 35-39 | 13 | 50 | 1 | 0 | 0.664 | 33 |
| 40-44 | 8 | 18 | 0 | 0 | 0.802 | 14 |
| 45-49 | 2 | 14 | 0 | 0 | 0.900 | 13 |
| **Total** | 87 | 252 | **6** | **1** |  | 126 |

* Adjusted number of sisters by multiplying the average number of sisters for respondents aged 25–49 (i.e., 1.64) by the number of respondents (age group 15–19 and 20–24). Originally the number of sisters was 34 for the age 15–19 years, and 73 for 20–24 years. LTR = 1/126 = 0.008, TFR = 5.4 for 7-previous years, and MM Ratio = 1-(1- LTR)^1/TFR^ = 149/100,000 LB (95%CI: -148, 444).

**Wealth quintile**

**Lowest**

| **Age of respondents**  **in years** | **No of respondents** | **No. of sisters survived (>15 years)** | **No. of sisters died from all maternal causes** | **No. of pregnancy-related deaths** | **Adjustment**  **factors** | **Sister unit of exposure (E)** |
| --- | --- | --- | --- | --- | --- | --- |
|  |  | **(A)** | **(B)** | **(C)** | **(D)** | **(E=A*D)** |
| 15-19 | 645 | 774* | 18 | 2 | 0.107 | 83 |
| 20-24 | 391 | 469* | 23 | 11 | 0.206 | 97 |
| 25-29 | 436 | 586 | 13 | 3 | 0.343 | 201 |
| 30-34 | 385 | 529 | 21 | 6 | 0.503 | 266 |
| 35-39 | 356 | 493 | 19 | 5 | 0.664 | 327 |
| 40-44 | 313 | 328 | 11 | 2 | 0.802 | 263 |
| 45-49 | 299 | 217 | 5 | 0 | 0.900 | 195 |
| **Total** | **2825** | **3396** | **100** | **29** |  | **1432** |

* Adjusted number of sisters by multiplying the average number of sisters for respondents aged 25–49 (i.e., 1.20) by the number of respondents (age group 15–19 and 20–24). Originally the number of sisters was 506 for the age 15–19 years, and 668 for 20–24 years.

LTR = 29/1432 = 0.020, TFR = 5.4 for 7-prevous years, and MM Ratio = 1-(1- LTR)^1/TFR^ = 373/100,000 LB (95%CI: 236, 505).

**Second**

| **Age of respondents**  **in years** | **No of respondents** | **No. of sisters survived (>15 years)** | **No. of sisters died from all maternal causes** | **No. of pregnancy-related deaths** | **Adjustment**  **factors** | **Sister unit of exposure (E)** |
| --- | --- | --- | --- | --- | --- | --- |
|  |  | **(A)** | **(B)** | **(C)** | **(D)** | **(E=A*D)** |
| 15-19 | 597 | 752* | 28 | 10 | 0.107 | 80 |
| 20-24 | 564 | 710* | 29 | 10 | 0.206 | 146 |
| 25-29 | 480 | 634 | 36 | 15 | 0.343 | 217 |
| 30-34 | 379 | 549 | 33 | 9 | 0.503 | 276 |
| 35-39 | 314 | 458 | 22 | 10 | 0.664 | 304 |
| 40-44 | 304 | 321 | 17 | 2 | 0.802 | 257 |
| 45-49 | 243 | 204 | 9 | 0 | 0.900 | 184 |
| **Total** | **2881** | **3628** | **174** | **56** |  | **1465** |

* Adjusted number of sisters by multiplying the average number of sisters for respondents aged 25–49 (i.e., 1.26) by the number of respondents (age group 15–19 and 20–24). Originally the number of sisters was 555 for the age 15–19 years, and 701 for 20–24 years.

LTR = 56/1465 = 0.038, TFR = 5.4 for 7-previous years, and MM Ratio= 1-(1- LTR)^1/TFR^ = 715/100,000 LB (95%CI: 522, 885).

**Middle**

| **Age of respondents**  **in years** | **No of respondents** | **No. of sisters survived (>15 years)** | **No. of sisters died from all maternal causes** | **No. of pregnancy-related deaths** | **Adjustment**  **factors** | **Sister unit of exposure (E)** |
| --- | --- | --- | --- | --- | --- | --- |
|  |  | **(A)** | **(B)** | **(C)** | **(D)** | **(E=A*D)** |
| 15-19 | 680 | 1054* | 32 | 5 | 0.107 | 113 |
| 20-24 | 615 | 953* | 39 | 9 | 0.206 | 196 |
| 25-29 | 513 | 762 | 37 | 15 | 0.343 | 261 |
| 30-34 | 358 | 689 | 31 | 12 | 0.503 | 347 |
| 35-39 | 319 | 524 | 24 | 3 | 0.664 | 348 |
| 40-44 | 253 | 362 | 14 | 4 | 0.802 | 290 |
| 45-49 | 219 | 239 | 17 | 0 | 0.900 | 215 |
| **Total** | **2957** | **4583** | **194** | **48** |  | **1770** |

* Adjusted number of sisters obtained by multiplying the average number of sisters for respondents aged 25–49 (i.e., 1.55) by the number of respondents (age group 15–19 and 20–24). Originally the number of sisters was 703 for the age 15–19 years, and 820 for 20–24 years. LTR = 48/1770 = 0.027, TFR = 5.4 for 7-previous years, and MM Ratio = 1-(1- LTR)^1/TFR^ = 506/100,000 LB (95%CI: 359, 641).

**Fourth**

| **Age of respondents**  **in years** | **No of respondents** | **No. of sisters survived (>15 years)** | **No. of sisters died from all maternal causes** | **No. of pregnancy-related deaths** | **Adjustment**  **Factors** | **Sister unit of exposure (E)** |
| --- | --- | --- | --- | --- | --- | --- |
|  |  | **(A)** | **(B)** | **(C)** | **(D)** | **(E=A*D)** |
| 15-19 | 739 | 1042* | 39 | 10 | 0.107 | 111 |
| 20-24 | 559 | 788* | 38 | 11 | 0.206 | 162 |
| 25-29 | 507 | 735 | 44 | 16 | 0.343 | 252 |
| 30-34 | 321 | 640 | 38 | 18 | 0.503 | 322 |
| 35-39 | 352 | 483 | 28 | 6 | 0.664 | 321 |
| 40-44 | 262 | 364 | 17 | 5 | 0.802 | 292 |
| 45-49 | 273 | 200 | 8 | 1 | 0.900 | 180 |
| **Total** | **3013** | **4252** | **212** | **67** |  | **1640** |

* Adjusted number of sisters by multiplying the average number of sisters for respondents aged 25–49 (i.e., 1.41) by the number of respondents (age group 15–19 and 20–24). Originally the number of sisters was 639 for the age 15–19 years, and 803 for 20–24 years.

LTR = 67/1640 = 0.041, TFR = 5.4 for 7-prevous years, and MM Ratio = 1-(1- LTR)^1/TFR^ = 772/100,000 LB (95%CI: 582, 937).

**Highest**

| **Age of respondents**  **in years** | **No of respondents** | **No. of sisters survived (>15 years)** | **No. of sisters died from all maternal causes** | **No. of pregnancy-related deaths** | **Adjustment**  **factors** | **Sister unit of exposure (E)** |
| --- | --- | --- | --- | --- | --- | --- |
|  |  | **(A)** | **(B)** | **(C)** | **(D)** | **(E=A*D)** |
| 15-19 | 1040 | 2829* | 47 | 6 | 0.107 | 303 |
| 20-24 | 727 | 1978* | 85 | 16 | 0.206 | 407 |
| 25-29 | 649 | 1786 | 80 | 12 | 0.343 | 613 |
| 30-34 | 396 | 1372 | 50 | 7 | 0.503 | 690 |
| 35-39 | 374 | 1004 | 36 | 5 | 0.664 | 667 |
| 40-44 | 259 | 660 | 33 | 2 | 0.802 | 529 |
| 45-49 | 230 | 372 | 25 | 4 | 0.900 | 335 |
| **Total** | **3675** | **10001** | **353** | **52** |  | **3544** |

* Adjusted number of sisters by multiplying the average number of sisters for respondents aged 25–49 (i.e., 2.72) by the number of respondents (age group 15–19 and 20–24). Originally the number of sisters was 1590 for the age 15–19 years, and 1977 for 20–24 years. LTR = 52/3544 = 0.015, TFR = 5.4 for 7-previous years, and MM Ratio = 1-(1- LTR)^1/TFR^ = 280/100,000 LB (95%CI: 204, 350).

**Sub-national administrative regions**

**Tigray**

| **Age of respondents**  **in years** | **No of respondents** | **No. of sisters survived (>15 years)** | **No. of sisters died from all maternal causes** | **No. of pregnancy-related deaths** | **Adjustment**  **factors** | **Sister unit of exposure (E)** |
| --- | --- | --- | --- | --- | --- | --- |
|  |  | **(A)** | **(B)** | **(C)** | **(D)** | **(E=A*D)** |
| 15-19 | 234 | 487* | 8 | 3 | 0.107 | 52 |
| 20-24 | 157 | 327* | 14 | 8 | 0.206 | 67 |
| 25-29 | 170 | 335 | 14 | 2 | 0.343 | 115 |
| 30-34 | 118 | 324 | 15 | 1 | 0.503 | 163 |
| 35-39 | 112 | 247 | 11 | 2 | 0.664 | 164 |
| 40-44 | 96 | 196 | 8 | 1 | 0.802 | 157 |
| 45-49 | 83 | 105 | 5 | 0 | 0.900 | 95 |
| **Total** | **970** | **2020** | **75** | **17** |  | **813** |

*Adjusted number of sisters by multiplying the average number of sisters for respondents aged 25–49 (i.e., 2.08) by the number of respondents (age group 15–19 and 20–24). Originally the number of sisters was 287 for the age 15–19 years, and 349 for 20–24 years.

LTR = 17/813 = 0.021, TFR = 5.4 for 7-previous years, and MM Ratio = 1-(1- LTR)^1/TFR^ = 392/100,000 LB (95%CI: 207, 571).

**Afar**

| **Age of respondents**  **in years** | **No of respondents** | **No. of sisters survived (>15 years)** | **No. of sisters died from all maternal causes** | **No. of pregnancy-related deaths** | **Adjustment**  **factors** | **Sister unit of exposure (E)** |
| --- | --- | --- | --- | --- | --- | --- |
|  |  | **(A)** | **(B)** | **(C)** | **(D)** | **(E=A*D)** |
| 15-19 | 34 | 175* | 14 | 3 | 0.107 | 19 |
| 20-24 | 30 | 154* | 13 | 5 | 0.206 | 32 |
| 25-29 | 31 | 169 | 16 | 3 | 0.343 | 58 |
| 30-34 | 25 | 134 | 18 | 2 | 0.503 | 67 |
| 35-39 | 23 | 133 | 12 | 3 | 0.664 | 88 |
| 40-44 | 18 | 82 | 6 | 2 | 0.802 | 66 |
| 45-49 | 17 | 68 | 9 | 0 | 0.900 | 61 |
| **Total** | **178** | **915** | **88** | **18** |  | **391** |

* Adjusted number of sisters by multiplying the average number of sisters for respondents aged 25–49 (i.e., 5.14) by the number of respondents (age group 15–19 and 20–24). Originally the number of sisters was 135 for the age 15–19 years, and 173 for 20–24 years.

LTR = 18/391 = 0.046, TFR = 5.4 for 7-previous years, and MM Ratio = 1-(1- LTR)^1/TFR^ =868/100,000 LB (95%CI: 467, 1237).

**Amhara**

| **Age of respondents**  **in years** | **No of respondents** | **No. of sisters survived (>15 years)** | **No. of sisters died from all maternal causes** | **No. of pregnancy-related deaths** | **Adjustment**  **factors** | **Sister unit of exposure (E)** |
| --- | --- | --- | --- | --- | --- | --- |
|  |  | **(A)** | **(B)** | **(C)** | **(D)** | **(E=A*D)** |
| 15-19 | 842 | 648* | 23 | 5 | 0.107 | 69 |
| 20-24 | 646 | 497* | 21 | 7 | 0.206 | 102 |
| 25-29 | 650 | 505 | 26 | 8 | 0.343 | 173 |
| 30-34 | 449 | 459 | 26 | 8 | 0.503 | 231 |
| 35-39 | 504 | 395 | 22 | 3 | 0.664 | 262 |
| 40-44 | 351 | 259 | 12 | 2 | 0.802 | 208 |
| 45-49 | 378 | 168 | 12 | 2 | 0.900 | 151 |
| **Total** | **3820** | **2032** | **142** | **35** |  | **1197** |

* Adjusted number of sisters by multiplying the average number of sisters for respondents aged 25–49 (i.e., 0.77) by the number of respondents (age group 15–19 and 20–24). Originally the number of sisters was 477 for the age 15–19 years, and 554 for 20–24 years.

LTR = 35/1197 = 0.029, TFR = 5.4 for 7-previous years, and MM Ratio = 1-(1- LTR)^1/TFR^ = 544/100,000 LB (95%CI: 361, 713).

**Oromiya**

| **Age of respondents**  **in years** | **No of respondents** | **No. of sisters survived (>15 years)** | **No. of sisters died from all maternal causes** | **No. of pregnancy-related deaths** | **Adjustment**  **factors** | **Sister unit of exposure (E)** |
| --- | --- | --- | --- | --- | --- | --- |
|  |  | **(A)** | **(B)** | **(C)** | **(D)** | **(E=A*D)** |
| 15-19 | 1594 | 1291* | 29 | 6 | 0.107 | 138 |
| 20-24 | 1180 | 956* | 39 | 12 | 0.206 | 197 |
| 25-29 | 931 | 813 | 28 | 8 | 0.343 | 279 |
| 30-34 | 708 | 635 | 30 | 14 | 0.503 | 319 |
| 35-39 | 575 | 527 | 26 | 8 | 0.664 | 350 |
| 40-44 | 520 | 351 | 17 | 2 | 0.802 | 282 |
| 45-49 | 428 | 234 | 5 | 0 | 0.900 | 211 |
| **Total** | **5936** | **4807** | **174** | **50** |  | **1775** |

* Adjusted number of sisters by multiplying the average number of sisters for respondents aged 25–49 (i.e., 0.81) by the number of respondents (age group 15–19 and 20–24). Originally the number of sisters was 761 for the age 15–19 years, and 960 for 20–24 years.

LTR = 50/1775 = 0.028, TFR = 5.4 for 7-previous years, and MM Ratio = 1-(1- LTR)^1/TFR^ = 525/100,000 LB (95%CI: 376, 661).

**Somali**

| **Age of respondents**  **in years** | **No of respondents** | **No. of sisters survived (>15 years)** | **No. of sisters died from all maternal causes** | **No. of pregnancy-related deaths** | **Adjustment**  **factors** | **Sister unit of exposure (E)** |
| --- | --- | --- | --- | --- | --- | --- |
|  |  | **(A)** | **(B)** | **(C)** | **(D)** | **(E=A*D)** |
| 15-19 | 43 | 369* | 10 | 1 | 0.107 | 40 |
| 20-24 | 27 | 232* | 7 | 3 | 0.206 | 48 |
| 25-29 | 27 | 275 | 10 | 4 | 0.343 | 94 |
| 30-34 | 28 | 257 | 9 | 5 | 0.503 | 129 |
| 35-39 | 26 | 185 | 6 | 4 | 0.664 | 123 |
| 40-44 | 12 | 118 | 3 | 0 | 0.802 | 95 |
| 45-49 | 12 | 67 | 2 | 0 | 0.900 | 60 |
| **Total** | **175** | **1503** | **47** | **17** |  | **589** |

* Adjusted number of sisters by multiplying the average number of sisters for respondents aged 25–49 (i.e., 8.59) by the number of respondents (age group 15–19 and 20–24). Originally the number of sisters was 206 for the age 15–19 years, and 277 for 20–24 years.

LTR = 17/589 = 0.029, TFR = 5.4 for 7-previous years, and MM Ratio = 1-(1- LTR)^1/TFR^ = 544/100,000 LB (95%CI: 287, 787).

**Beshngul Gumuz**

| **Age of respondents**  **in years** | **No of respondents** | **No. of sisters survived (>15 years)** | **No. of sisters died from all maternal causes** | **No. of pregnancy-related deaths** | **Adjustment**  **factors** | **Sister unit of exposure (E)** |
| --- | --- | --- | --- | --- | --- | --- |
|  |  | **(A)** | **(B)** | **(C)** | **(D)** | **(E=A*D)** |
| 15-19 | 41 | 372* | 15 | 5 | 0.107 | 40 |
| 20-24 | 27 | 245* | 13 | 2 | 0.206 | 50 |
| 25-29 | 26 | 264 | 13 | 6 | 0.343 | 91 |
| 30-34 | 22 | 215 | 9 | 6 | 0.503 | 108 |
| 35-39 | 21 | 139 | 10 | 2 | 0.664 | 92 |
| 40-44 | 10 | 131 | 5 | 2 | 0.802 | 105 |
| 45-49 | 12 | 76 | 1 | 0 | 0.900 | 68 |
| **Total** | **159** | **1442** | **66** | **23** |  | **555** |

* Adjusted number of sisters by multiplying the average number of sisters for respondents aged 25–49 (i.e., 9.07) by the number of respondents (age group 15–19 and 20–24). Originally the number of sisters was 265 for the age 15–19 years, and 297 for 20–24 years.

LTR = 23/555 = 0.042, TFR = 5.4 for 7-previous years, and MM Ratio = 1-(1- LTR)^1/TFR^ = 791/100,000 LB (95%CI: 469, 1087).

**SNNP**

| **Age of respondents**  **in years** | **No of respondents** | **No. of sisters survived (>15 years)** | **No. of sisters died from all maternal causes** | **No. of pregnancy-related deaths** | **Adjustment**  **factors** | **Sister unit of exposure (E)** |
| --- | --- | --- | --- | --- | --- | --- |
|  |  | **(A)** | **(B)** | **(C)** | **(D)** | **(E=A*D)** |
| 15-19 | 688 | 716* | 8 | 2 | 0.107 | 77 |
| 20-24 | 622 | 647* | 26 | 8 | 0.206 | 133 |
| 25-29 | 591 | 588 | 20 | 9 | 0.343 | 202 |
| 30-34 | 408 | 517 | 21 | 6 | 0.503 | 260 |
| 35-39 | 370 | 446 | 11 | 4 | 0.664 | 296 |
| 40-44 | 323 | 299 | 8 | 2 | 0.802 | 240 |
| 45-49 | 282 | 184 | 6 | 0 | 0.900 | 166 |
| **Total** | **3284** | **3396** | **100** | **31** |  | **1373** |

* Adjusted number of sisters by multiplying the average number of sisters for respondents aged 25–49 (i.e., 1.03) by the number of respondents (age group 15–19 and 20–24). Originally the number of sisters was 517 for the age 15–19 years, and 664 for 20–24 years.

LTR = 31/1373 = 0.023, TFR = 5.4 for 7-previous years, and MM Ratio = 1-(1- LTR)^1/TFR^ = 430/100,000 LB (95%CI: 280, 572).

**Gambella**

| **Age of respondents**  **in years** | **No of respondents** | **No. of sisters survived (>15 years)** | **No. of sisters died from all maternal causes** | **No. of pregnancy-related deaths** | **Adjustment**  **factors** | **Sister unit of exposure (E)** |
| --- | --- | --- | --- | --- | --- | --- |
|  |  | **(A)** | **(B)** | **(C)** | **(D)** | **(E=A*D)** |
| 15-19 | 8 | 221* | 12 | 3 | 0.107 | 24 |
| 20-24 | 8 | 221* | 19 | 4 | 0.206 | 45 |
| 25-29 | 9 | 192 | 14 | 6 | 0.343 | 66 |
| 30-34 | 6 | 181 | 10 | 2 | 0.503 | 91 |
| 35-39 | 4 | 134 | 9 | 0 | 0.664 | 89 |
| 40-44 | 2 | 81 | 7 | 3 | 0.802 | 65 |
| 45-49 | 2 | 46 | 3 | 0 | 0.900 | 41 |
| **Total** | **39** | **1075** | **74** | **18** |  | **421** |

* Adjusted number of sisters by multiplying the average number of sisters for respondents aged 25–49 (i.e., 27.57) by the number of respondents (age group 15–19 and 20–24). Originally the number of sisters was 180 for the age 15–19 years, and 211 for 20–24 years.

LTR = 18/421 = 0.043, TFR = 5.4 for 7-previous years, and MM Ratio = 1-(1- LTR)^1/TFR^ = 811/100,000 LB (95%CI: 437, 1155).

**Harari**

| **Age of respondents**  **in years** | **No of respondents** | **No. of sisters survived (>15 years)** | **No. of sisters died from all maternal causes** | **No. of pregnancy-related deaths** | **Adjustment**  **Factors** | **Sister unit of exposure (E)** |
| --- | --- | --- | --- | --- | --- | --- |
|  |  | **(A)** | **(B)** | **(C)** | **(D)** | **(E=A*D)** |
| 15-19 | 9 | 360* | 7 | 0 | 0.107 | 39 |
| 20-24 | 9 | 360* | 17 | 2 | 0.206 | 74 |
| 25-29 | 7 | 324 | 20 | 4 | 0.343 | 111 |
| 30-34 | 5 | 225 | 8 | 3 | 0.503 | 113 |
| 35-39 | 5 | 151 | 2 | 1 | 0.664 | 100 |
| 40-44 | 3 | 145 | 4 | 2 | 0.802 | 116 |
| 45-49 | 3 | 76 | 4 | 0 | 0.900 | 68 |
| **Total** | **41** | **1642** | **62** | **12** |  | **622** |

* Adjusted number of sisters by multiplying the average number of sisters for respondents aged 25–49 (i.e., 40.04) by the number of respondents (age group 15–19 and 20–24). Originally the number of sisters was 231 for the age 15–19 years, and 338 for 20–24 years.

LTR = 12/622 = 0.019, TFR = 5.4 for 7-previous years, and MM Ratio = 1-(1- LTR)^1/TFR^ = 355/100,000 LB (95%CI: 152, 552).

**Addis Ababa**

| **Age of respondents**  **in years** | **No of respondents** | **No. of sisters survived (>15 years)** | **No. of sisters died from all maternal causes** | **No. of pregnancy-related deaths** | **Adjustment**  **Factors** | **Sister unit of exposure (E)** |
| --- | --- | --- | --- | --- | --- | --- |
|  |  | **(A)** | **(B)** | **(C)** | **(D)** | **(E=A*D)** |
| 15-19 | 199 | 1128* | 21 | 3 | 0.107 | 121 |
| 20-24 | 136 | 771* | 27 | 3 | 0.206 | 159 |
| 25-29 | 126 | 696 | 37 | 3 | 0.343 | 239 |
| 30-34 | 63 | 534 | 22 | 3 | 0.503 | 269 |
| 35-39 | 69 | 387 | 14 | 1 | 0.664 | 257 |
| 40-44 | 48 | 221 | 14 | 0 | 0.802 | 177 |
| 45-49 | 43 | 140 | 10 | 3 | 0.900 | 126 |
| **Total** | **684** | **3877** | **145** | **16** |  | **1347** |

* Adjusted number of sisters by multiplying the average number of sisters for respondents aged 25–49 (i.e., 5.67) by the number of respondents (age group 15–19 and 20–24). Originally the number of sisters was 644 for the age 15–19 years, and 777 for 20–24 years.

LTR = 16/1347 = 0.012, TFR = 5.4 for 7-previous years, and MM Ratio = 1-(1- LTR)^1/TFR^ = 223/100,000 LB (95%CI: 113, 332).

**Dire Dawa**

| **Age of respondents**  **in years** | **No of respondents** | **No. of sisters survived (>15 years)** | **No. of sisters died from all maternal causes** | **No. of pregnancy-related deaths** | **Adjustment**  **Factors** | **Sister unit of exposure (E)** |
| --- | --- | --- | --- | --- | --- | --- |
|  |  | **(A)** | **(B)** | **(C)** | **(D)** | **(E=A*D)** |
| 15-19 | 18 | 426* | 7 | 3 | 0.107 | 46 |
| 20-24 | 16 | 379* | 16 | 3 | 0.206 | 78 |
| 25-29 | 17 | 346 | 12 | 4 | 0.343 | 119 |
| 30-34 | 10 | 303 | 5 | 2 | 0.503 | 152 |
| 35-39 | 8 | 217 | 6 | 1 | 0.664 | 144 |
| 40-44 | 6 | 154 | 8 | 0 | 0.802 | 124 |
| 45-49 | 5 | 69 | 7 | 1 | 0.900 | 62 |
| **Total** | **80** | **1894** | **61** | **14** |  | **724** |

* Adjusted number of sisters by multiplying the average number of sisters for respondents aged 25–49 (i.e., 23.67) by the number of respondents (age group 15–19 and 20–24). Originally the number of sisters was 297 for the age 15–19 years, and 374 for 20–24 years.

LTR = 14/724 = 0.019, TFR = 5.4 for 7-previous years, and MM Ratio = 1-(1- LTR)^1/TFR^ = 355/100,000 LB (95%CI: 168, 535).
