# Supplementary material for: A woman's lifetime risk disparities in maternal mortality in Ethiopia
Source: Public Health Chall. 2023 Jan 13;2(1):e56. doi: 10.1002/puh2.56 (PMC12039551; doi:10.1002/puh2.56)
Supplement: Supplementary file 2 — Estimation of lifetime risks of maternal mortality for the year 2005, Ethiopia [file PUH2-2-e56-s003.docx]

**Supplementary file 2.** Estimation of lifetime risks of maternal mortality for the year 2005, Ethiopia

**Place of residence**

**Urban**

| **Age of respondents**  **in years** | **No of respondents** | **No. of sisters survived**  **(>15 years)** | **No. of sisters died from all maternal causes** | **No. of pregnancy-related deaths** | **Adjustment**  **factors** | **Sister unit of exposure (E)** |
| --- | --- | --- | --- | --- | --- | --- |
|  |  | **(A)** | **(B)** | **(C)** | **(D)** | **(E=A*D)** |
| 15-19 | 703 | 1287* | 5 | 0 | 0.107 | 138 |
| 20-24 | 539 | 986* | 29 | 9 | 0.206 | 203 |
| 25-29 | 422 | 787 | 34 | 5 | 0.343 | 270 |
| 30-34 | 252 | 582 | 17 | 0 | 0.503 | 293 |
| 35-39 | 232 | 455 | 15 | 0 | 0.664 | 302 |
| 40-44 | 177 | 288 | 13 | 0 | 0.802 | 231 |
| 45-49 | 173 | 181 | 6 | 0 | 0.900 | 163 |
| **Total** | 2498 | 4566 | **119** | **14** |  | 1600 |

* Adjusted number of sisters by multiplying the average number of sisters for respondents aged 25–49 (i.e., 1.83) by the number of respondents (age group 15–19 and 20–24). Originally the number of sisters was 794 for the age 15–19 years, and 978 for 20–24 years.

LTR = 14/1600 = 0.009, TFR = 5.4 for 3-previous years, and MM Ratio = 1-(1- LTR)^1/TFR^ = 167/100,000 LB (95%CI: 80, 254).

**Rural**

| **Age of respondents**  **in years** | **No of respondents** | **No. of sisters survived (>15 years)** | **No. of sisters died from all maternal causes** | **No. of pregnancy-related deaths** | **Adjustment**  **Factors** | **Sister unit of exposure (E)** |
| --- | --- | --- | --- | --- | --- | --- |
|  |  | **(A)** | **(B)** | **(C)** | **(D)** | **(E=A*D)** |
| 15-19 | 2562 | 4330* | 103 | 14 | 0.107 | 463 |
| 20-24 | 2008 | 3807^ | 130 | 38 | 0.206 | 784 |
| 25-29 | 2095 | 3476 | 129 | 47 | 0.343 | 1192 |
| 30-34 | 1555 | 3212 | 152 | 47 | 0.503 | 1616 |
| 35-39 | 1370 | 2466 | 105 | 34 | 0.664 | 1637 |
| 40-44 | 1010 | 1685 | 54 | 5 | 0.802 | 1351 |
| 45-49 | 970 | 956 | 47 | 1 | 0.900 | 860 |
| **Total** | 11570 | 19932 | **720** | **186** |  | 7905 |

* Adjusted number of sisters by multiplying the average number of sisters for respondents aged 25–49 (i.e., 1.69) by the number of respondents (age group 15–19). Originally the number of sisters was 3198 for 15–19 years.

^ No adjustment. As the number of sisters after adjustment was much less than the number of sisters reported, the actual number of sisters was used. Because, after adjustment, the number of sisters was 3394 for the age 20–24 years.

LTR = 186/7905 = 0.024, TFR = 5.4 for 3-previous years, and MM Ratio = 1-(1- LTR)^1/TFR^ = 449/100,000 LB (95%CI: 383, 516).

**Educational status**

**No education**

| **Age of respondents**  **in years** | **No of respondents** | **No. of sisters survived (>15 years)** | **No. of sisters died from all maternal causes** | **No. of pregnancy-related deaths** | **Adjustment**  **factors** | **Sister unit of exposure (E)** |
| --- | --- | --- | --- | --- | --- | --- |
|  |  | **(A)** | **(B)** | **(C)** | **(D)** | **(E=A*D)** |
| 15-19 | 1308 | 2239^ | 68 | 11 | 0.107 | 240 |
| 20-24 | 1533 | 2715^ | 99 | 26 | 0.206 | 559 |
| 25-29 | 1734 | 2638 | 108 | 37 | 0.343 | 905 |
| 30-34 | 1319 | 2493 | 141 | 40 | 0.503 | 1254 |
| 35-39 | 1283 | 2165 | 83 | 29 | 0.664 | 1438 |
| 40-44 | 1033 | 1586 | 51 | 5 | 0.802 | 1272 |
| 45-49 | 1061 | 939 | 38 | 1 | 0.900 | 845 |
| **Total** | 9271 | 14775 | **588** | **149** |  | 6512 |

^ The adjustment factor was 1.53. However, there was no adjustment. As the number of sisters after adjustment was much less than the number of sisters reported, the actual number of sisters was used. Because, after adjustment, the number of sisters was 2001 for the age 15–19 years and 2346 for the age 20–24 years.

LTR = 149/6512 = 0.023, TFR = 5.4 for 3-previous years, and MM Ratio = 1-(1- LTR)^1/TFR^ = 430/100,000 LB (95%CI: 357, 495).

**Primary**

| **Age of respondents**  **in years** | **No of respondents** | **No. of sisters survived (>15 years)** | **No. of sisters died from all maternal causes** | **No. of pregnancy-related deaths** | **Adjustment**  **Factors** | **Sister unit of exposure (E)** |
| --- | --- | --- | --- | --- | --- | --- |
|  |  | **(A)** | **(B)** | **(C)** | **(D)** | **(E=A*D)** |
| 15-19 | 1423 | 3501 | 29 | 3 | 0.107 | 375 |
| 20-24 | 573 | 1410 | 42 | 17 | 0.206 | 290 |
| 25-29 | 479 | 1018 | 31 | 11 | 0.343 | 349 |
| 30-34 | 311 | 889 | 20 | 1 | 0.503 | 447 |
| 35-39 | 204 | 487 | 26 | 6 | 0.664 | 323 |
| 40-44 | 84 | 247 | 13 | 0 | 0.802 | 198 |
| 45-49 | 50 | 134 | 8 | 1 | 0.900 | 121 |
| **Total** | 3124 | 7686 | **169** | **39** |  | 2103 |

* Adjusted number of sisters by multiplying the average number of sisters for respondents aged 25–49 (i.e., 2.46) by the number of respondents (age group 15–19 and 20–24). Originally the number of sisters was 1135 for the age 15–19 years, and 1352 for 20–24 years. LTR = 39/2103 = 0.019, TFR = 5.4 for 3-previous years, and MM Ratio = 1-(1- LTR)^1/TFR^ = 355/100,000 LB (95%CI: 243, 461).

**Secondary**

| **Age of respondents**  **in years** | **No of respondents** | **No. of sisters survived (>15 years)** | **No. of sisters died from all maternal causes** | **No. of pregnancy-related deaths** | **Adjustment**  **Factors** | **Sister unit of exposure (E)** |
| --- | --- | --- | --- | --- | --- | --- |
|  |  | **(A)** | **(B)** | **(C)** | **(D)** | **(E=A*D)** |
| 15-19 | 519 | 1131* | 11 | 0 | 0.107 | 121 |
| 20-24 | 387 | 844* | 15 | 4 | 0.206 | 174 |
| 25-29 | 262 | 528 | 19 | 3 | 0.343 | 181 |
| 30-34 | 148 | 346 | 10 | 0 | 0.503 | 174 |
| 35-39 | 91 | 211 | 9 | 0 | 0.664 | 140 |
| 40-44 | 51 | 117 | 2 | 0 | 0.802 | 94 |
| 45-49 | 23 | 54 | 5 | 0 | 0.900 | 49 |
| **Total** | 1481 | 3231 | **71** | **7** |  | 933 |

* Adjusted number of sisters obtained by multiplying the average number of sisters for respondents aged 25–49 (i.e., 2.18) by the number of respondents (age group 15–19 and 20–24). Originally the number of sisters was 553 for the age 15–19 years, and 639 for 20–24 years. LTR = 7/933 = 0.008, TFR = 5.4 for 3-previous years, and MM Ratio = 1-(1- LTR)^1/TFR^ = 149/100,000 LB (95%CI: 43, 253).

**Higher**

| **Age of respondents**  **in years** | **No of respondents** | **No. of sisters survived (>15 years)** | **No. of sisters died from all maternal causes** | **No. of pregnancy-related deaths** | **Adjustment**  **Factors** | **Sister unit of exposure (E)** |
| --- | --- | --- | --- | --- | --- | --- |
|  |  | **(A)** | **(B)** | **(C)** | **(D)** | **(E=A*D)** |
| 15-19 | 16 | 66^ | 0 | 0 | 0.107 | 7 |
| 20-24 | 53 | 101* | 1 | 0 | 0.206 | 21 |
| 25-29 | 43 | 80 | 2 | 1 | 0.343 | 27 |
| 30-34 | 30 | 66 | 0 | 0 | 0.503 | 33 |
| 35-39 | 24 | 57 | 4 | 0 | 0.664 | 38 |
| 40-44 | 19 | 25 | 0 | 0 | 0.802 | 20 |
| 45-49 | 9 | 9 | 0 | 0 | 0.900 | 8 |
| **Total** | 194 | 404 | **7** | **1** |  | 155 |

^ No adjustment. As the number of sisters after adjustment was much less than the number of sisters reported, the actual number of sisters was used. Because, after adjustment, the number of sisters was 30 for the age 15–19 years. * Adjusted number of sisters by multiplying the average number of sisters for respondents aged 25–49 (i.e., 1.90) by the number of respondents (age group 20–24). Originally the number of sisters was 81 for 20–24 years.

LTR = 1/155 = 0.007, TFR = 5.4 for 3-previous years, and MM Ratio = 1-(1- LTR)^1/TFR^ = 130/100,000 LB (95%CI: 114, 373).

**Wealth quintile**

**Lowest**

| **Age of respondents**  **in years** | **No of respondents** | **No. of sisters survived (>15 years)** | **No. of sisters died from all maternal causes** | **No. of pregnancy-related deaths** | **Adjustment**  **factors** | **Sister unit of exposure (E)** |
| --- | --- | --- | --- | --- | --- | --- |
|  |  | **(A)** | **(B)** | **(C)** | **(D)** | **(E=A*D)** |
| 15-19 | 448 | 677* | 25 | 0 | 0.107 | 72 |
| 20-24 | 389 | 732^ | 36 | 10 | 0.206 | 151 |
| 25-29 | 431 | 699 | 19 | 8 | 0.343 | 240 |
| 30-34 | 381 | 633 | 27 | 7 | 0.503 | 318 |
| 35-39 | 321 | 510 | 12 | 8 | 0.664 | 339 |
| 40-44 | 241 | 370 | 7 | 0 | 0.802 | 297 |
| 45-49 | 217 | 192 | 0 | 0 | 0.900 | 173 |
| **Total** | 2428 | 3813 | **126** | **33** |  | 1590 |

* Adjusted number of sisters by multiplying the average number of sisters for respondents aged 25–49 (i.e., 1.51) by the number of respondents (age group 15–19). Originally the number of sisters was 571 for 15–19 years.

^ No adjustment. As the number of sisters after adjustment was much less than the number of sisters reported, the actual number of sisters was used. Because, after adjustment, the number of sisters was 587 for the age 20–24 years.

LTR = 33/1590 = 0.021, TFR = 5.4 for 3-previous years, and MM Ratio = 1-(1- LTR)^1/TFR^ = 392/100,000 LB (95%CI: 258, 464).

**Second**

| **Age of respondents**  **in years** | **No of respondents** | **No. of sisters survived (>15 years)** | **No. of sisters died from all maternal causes** | **No. of pregnancy-related deaths** | **Adjustment**  **factors** | **Sister unit of exposure (E)** |
| --- | --- | --- | --- | --- | --- | --- |
|  |  | **(A)** | **(B)** | **(C)** | **(D)** | **(E=A*D)** |
| 15-19 | 566 | 962* | 27 | 3 | 0.107 | 103 |
| 20-24 | 479 | 821^ | 22 | 4 | 0.206 | 169 |
| 25-29 | 431 | 779 | 34 | 12 | 0.343 | 267 |
| 30-34 | 367 | 742 | 39 | 15 | 0.503 | 373 |
| 35-39 | 336 | 578 | 28 | 9 | 0.664 | 384 |
| 40-44 | 246 | 372 | 13 | 0 | 0.802 | 298 |
| 45-49 | 218 | 241 | 14 | 1 | 0.900 | 217 |
| **Total** | 2643 | 4495 | **177** | **44** |  | 1812 |

* Adjusted number of sisters by multiplying the average number of sisters for respondents aged 25–49 (i.e., 1.70) by the number of respondents (age group 15–19). Originally the number of sisters was 672 for 15–19 years.

^ No adjustment. As the number of sisters after adjustment was much less than the number of sisters reported, the actual number of sisters was used. Because, after adjustment, the number of sisters was 814 for the age 20–24 years.

LTR = 44/1812 = 0.024, TFR = 5.4 for 3-previous years, and MM Ratio= 1-(1- LTR)^1/TFR^ = 449/100,000 LB (95%CI: 314, 575).

**Middle**

| **Age of respondents**  **in years** | **No of respondents** | **No. of sisters survived (>15 years)** | **No. of sisters died from all maternal causes** | **No. of pregnancy-related deaths** | **Adjustment**  **factors** | **Sister unit of exposure (E)** |
| --- | --- | --- | --- | --- | --- | --- |
|  |  | **(A)** | **(B)** | **(C)** | **(D)** | **(E=A*D)** |
| 15-19 | 627 | 1072* | 20 | 5 | 0.107 | 115 |
| 20-24 | 508 | 980^ | 25 | 10 | 0.206 | 202 |
| 25-29 | 554 | 863 | 30 | 13 | 0.343 | 296 |
| 30-34 | 353 | 717 | 32 | 11 | 0.503 | 361 |
| 35-39 | 278 | 595 | 20 | 0 | 0.664 | 395 |
| 40-44 | 203 | 362 | 10 | 2 | 0.802 | 290 |
| 45-49 | 209 | 198 | 16 | 0 | 0.900 | 178 |
| **Total** | 2732 | 4787 | **153** | **41** |  | 1837 |

* Adjusted number of sisters by multiplying the average number of sisters for respondents aged 25–49 (i.e., 1.71) by the number of respondents (age group 15–19). Originally the number of sisters was 847 for 15–19 years.

^ No adjustment. As the number of sisters after adjustment was much less than the number of sisters reported, the actual number of sisters was used. Because, after adjustment, the number of sisters was 869 for the age 20–24 years.

LTR = 41/1837 = 0.022, TFR = 5.4 for 3-previous years, and MM Ratio = 1-(1- LTR)^1/TFR^ = 411/100,000 LB (95%CI: 281, 535).

**Fourth**

| **Age of respondents**  **in years** | **No of respondents** | **No. of sisters survived (>15 years)** | **No. of sisters died from all maternal causes** | **No. of pregnancy-related deaths** | **Adjustment**  **Factors** | **Sister unit of exposure (E)** |
| --- | --- | --- | --- | --- | --- | --- |
|  |  | **(A)** | **(B)** | **(C)** | **(D)** | **(E=A*D)** |
| 15-19 | 603 | 1031* | 24 | 4 | 0.107 | 110 |
| 20-24 | 440 | 876^ | 26 | 7 | 0.206 | 180 |
| 25-29 | 514 | 759 | 34 | 10 | 0.343 | 260 |
| 30-34 | 306 | 776 | 32 | 8 | 0.503 | 390 |
| 35-39 | 291 | 561 | 27 | 10 | 0.664 | 373 |
| 40-44 | 241 | 412 | 17 | 1 | 0.802 | 330 |
| 45-49 | 251 | 238 | 10 | 0 | 0.900 | 214 |
| **Total** | 2646 | 4653 | **170** | **40** |  | 1859 |

* Adjusted number of sisters by multiplying the average number of sisters for respondents aged 25–49 (i.e., 1.71) by the number of respondents (age group 15–19). Originally the number of sisters was 773 for 15–19 years.

^ No adjustment. As the number of sisters after adjustment was much less than the number of sisters reported, the actual number of sisters was used. Because, after adjustment, the number of sisters was 752 for the age 20–24 years.

LTR = 40/1859 = 0.022, TFR = 5.4 for 3-previous years, and MM Ratio = 1-(1- LTR)^1/TFR^ = 411/100,000 LB (95%CI: 284, 531).

**Highest**

| **Age of respondents**  **in years** | **No of respondents** | **No. of sisters survived (>15 years)** | **No. of sisters died from all maternal causes** | **No. of pregnancy-related deaths** | **Adjustment**  **factors** | **Sister unit of exposure (E)** |
| --- | --- | --- | --- | --- | --- | --- |
|  |  | **(A)** | **(B)** | **(C)** | **(D)** | **(E=A*D)** |
| 15-19 | 1022 | 1911* | 12 | 1 | 0.107 | 204 |
| 20-24 | 731 | 1377^ | 52 | 15 | 0.206 | 284 |
| 25-29 | 586 | 1162 | 49 | 10 | 0.343 | 399 |
| 30-34 | 400 | 924 | 38 | 7 | 0.503 | 465 |
| 35-39 | 377 | 676 | 32 | 3 | 0.664 | 449 |
| 40-44 | 256 | 459 | 20 | 2 | 0.802 | 368 |
| 45-49 | 248 | 271 | 11 | 0 | 0.900 | 244 |
| **Total** | 3620 | 6780 | **214** | **38** |  | 2412 |

* Adjusted number of sisters by multiplying the average number of sisters for respondents aged 25–49 (i.e., 1.87) by the number of respondents (age group 15–19). Originally the number of sisters was 1128 for 15–19 years.

^ No adjustment. As the number of sisters after adjustment was much less than the number of sisters reported, the actual number of sisters was used. Because, after adjustment, the number of sisters was 1367 for the age 20–24 years.

LTR = 38/2412 = 0.016, TFR = 5.4 for 3-previous years, and MM Ratio = 1-(1- LTR)^1/TFR^ =298/100,000 LB (95%CI: 202, 391).

**Sub-national administrative regions**

**Tigray**

| **Age of respondents**  **in years** | **No of respondents** | **No. of sisters survived (>15 years)** | **No. of sisters died from all maternal causes** | **No. of pregnancy-related deaths** | **Adjustment**  **factors** | **Sister unit of exposure (E)** |
| --- | --- | --- | --- | --- | --- | --- |
|  |  | **(A)** | **(B)** | **(C)** | **(D)** | **(E=A*D)** |
| 15-19 | 229 | 366* | 4 | 0 | 0.107 | 39 |
| 20-24 | 158 | 288^ | 4 | 1 | 0.206 | 59 |
| 25-29 | 148 | 238 | 10 | 4 | 0.343 | 82 |
| 30-34 | 125 | 238 | 15 | 7 | 0.503 | 120 |
| 35-39 | 103 | 180 | 8 | 0 | 0.664 | 120 |
| 40-44 | 77 | 124 | 2 | 1 | 0.802 | 99 |
| 45-49 | 80 | 72 | 2 | 0 | 0.900 | 65 |
| **Total** | 920 | 1506 | **45** | **13** |  | 584 |

* Adjusted number of sisters by multiplying the average number of sisters for respondents aged 25–49 (i.e., 1.60) by the number of respondents (age group 15–19). Originally the number of sisters was 208 for 15–19 years.

^ No adjustment. As the number of sisters after adjustment was much less than the number of sisters reported, the actual number of sisters was used. Because, after adjustment, the number of sisters was 253 for the age 20–24 years.

LTR = 13/584 = 0.023, TFR = 5.4 for 3-previous years, and MM Ratio = 1-(1- LTR)^1/TFR^ = 430/100,000 LB (95%CI: 201, 651).

**Afar**

| **Age of respondents**  **in years** | **No of respondents** | **No. of sisters survived (>15 years)** | **No. of sisters died from all maternal causes** | **No. of pregnancy-related deaths** | **Adjustment**  **factors** | **Sister unit of exposure (E)** |
| --- | --- | --- | --- | --- | --- | --- |
|  |  | **(A)** | **(B)** | **(C)** | **(D)** | **(E=A*D)** |
| 15-19 | 31 | 43* | 0 | 0 | 0.107 | 5 |
| 20-24 | 23 | 38^ | 1 | 0 | 0.206 | 8 |
| 25-29 | 24 | 41 | 1 | 1 | 0.343 | 14 |
| 30-34 | 20 | 29 | 0 | 0 | 0.503 | 15 |
| 35-39 | 20 | 25 | 0 | 0 | 0.664 | 17 |
| 40-44 | 15 | 19 | 0 | 0 | 0.802 | 15 |
| 45-49 | 12 | 13 | 0 | 0 | 0.900 | 12 |
| **Total** | 145 | 208 | **2** | **1** |  | 85 |

* Adjusted number of sisters by multiplying the average number of sisters for respondents aged 25–49 (i.e., 1.40) by the number of respondents (age group 15–19). Originally the number of sisters was 32 for 15–19 years.

^ No adjustment. As the number of sisters after adjustment was much less than the number of sisters reported, the actual number of sisters was used. Because, after adjustment, the number of sisters was 32 for the age 20–24 years.

LTR = 1/85 = 0.012, TFR = 5.4 for 3-previous years, and MM Ratio = 1-(1- LTR)^1/TFR^ = 223/100,000 LB (95%CI: -207, 650).

**Amhara**

| **Age of respondents**  **in years** | **No of respondents** | **No. of sisters survived (>15 years)** | **No. of sisters died from all maternal causes** | **No. of pregnancy-related deaths** | **Adjustment**  **factors** | **Sister unit of exposure (E)** |
| --- | --- | --- | --- | --- | --- | --- |
|  |  | **(A)** | **(B)** | **(C)** | **(D)** | **(E=A*D)** |
| 15-19 | 811 | 1265* | 31 | 5 | 0.107 | 135 |
| 20-24 | 582 | 1054^ | 48 | 11 | 0.206 | 217 |
| 25-29 | 589 | 925 | 54 | 9 | 0.343 | 317 |
| 30-34 | 425 | 867 | 59 | 18 | 0.503 | 436 |
| 35-39 | 418 | 714 | 33 | 7 | 0.664 | 474 |
| 40-44 | 328 | 463 | 31 | 4 | 0.802 | 371 |
| 45-49 | 329 | 281 | 26 | 0 | 0.900 | 253 |
| **Total** | 3482 | 5569 | **282** | **54** |  | 2204 |

* Adjusted number of sisters by multiplying the average number of sisters for respondents aged 25–49 (i.e., 1.56) by the number of respondents (age group 15–19). Originally the number of sisters was 875 for 15–19 years.

^ No adjustment. As the number of sisters after adjustment was much less than the number of sisters reported, the actual number of sisters was used. Because, after adjustment, the number of sisters was 908 for the age 20–24 years.

LTR = 54/2204 = 0.025, TFR = 5.4 for 3-previous years, and MM Ratio = 1-(1- LTR)^1/TFR^ = 468/100,000 LB (95%CI: 340, 587).

**Oromiya**

| **Age of respondents**  **in years** | **No of respondents** | **No. of sisters survived (>15 years)** | **No. of sisters died from all maternal causes** | **No. of pregnancy-related deaths** | **Adjustment**  **factors** | **Sister unit of exposure (E)** |
| --- | --- | --- | --- | --- | --- | --- |
|  |  | **(A)** | **(B)** | **(C)** | **(D)** | **(E=A*D)** |
| 15-19 | 1206 | 2195* | 38 | 4 | 0.107 | 235 |
| 20-24 | 925 | 1799^ | 57 | 18 | 0.206 | 371 |
| 25-29 | 879 | 1595 | 36 | 13 | 0.343 | 547 |
| 30-34 | 633 | 1426 | 64 | 14 | 0.503 | 717 |
| 35-39 | 550 | 1069 | 47 | 23 | 0.664 | 710 |
| 40-44 | 403 | 718 | 23 | 0 | 0.802 | 576 |
| 45-49 | 414 | 429 | 9 | 0 | 0.900 | 386 |
| **Total** | 5010 | 9231 | **274** | **72** |  | 3542 |

* Adjusted number of sisters by multiplying the average number of sisters for respondents aged 25–49 (i.e., 1.82) by the number of respondents (age group 15–19). Originally the number of sisters was 1627 for 15–19 years.

^ No adjustment. As the number of sisters after adjustment was much less than the number of sisters reported, the actual number of sisters was used. Because, after adjustment, the number of sisters was 1684 for the age 20–24 years.

LTR = 72/3542 = 0.021, TFR = 5.4 for 3-previous years, and MM Ratio = 1-(1- LTR)^1/TFR^ = 392/100,000 LB (95%CI: 302, 476).

**Somali**

| **Age of respondents**  **in years** | **No of respondents** | **No. of sisters survived (>15 years)** | **No. of sisters died from all maternal causes** | **No. of pregnancy-related deaths** | **Adjustment**  **factors** | **Sister unit of exposure (E)** |
| --- | --- | --- | --- | --- | --- | --- |
|  |  | **(A)** | **(B)** | **(C)** | **(D)** | **(E=A*D)** |
| 15-19 | 78 | 126* | 2 | 1 | 0.107 | 13 |
| 20-24 | 77 | 158^ | 5 | 2 | 0.206 | 33 |
| 25-29 | 108 | 149 | 7 | 4 | 0.343 | 51 |
| 30-34 | 87 | 139 | 2 | 0 | 0.503 | 70 |
| 35-39 | 64 | 128 | 1 | 0 | 0.664 | 85 |
| 40-44 | 48 | 77 | 0 | 0 | 0.802 | 62 |
| 45-49 | 25 | 43 | 0 | 0 | 0.900 | 39 |
| **Total** | 487 | 820 | **17** | **7** |  | 353 |

* Adjusted number of sisters by multiplying the average number of sisters for respondents aged 25–49 (i.e., 1.61) by the number of respondents (age group 15–19). Originally the number of sisters was 113 for 15–19 years.

^ No adjustment. As the number of sisters after adjustment was much less than the number of sisters reported, the actual number of sisters was used. Because, after adjustment, the number of sisters was 124 for the age 20–24 years.

LTR = 7/353 = 0.020, TFR = 5.4 for 3-previous years, and MM Ratio = 1-(1- LTR)^1/TFR^ = 374/100,000 LB (95%CI: 98, 643).

**Beshngul Gumuz**

| **Age of respondents**  **in years** | **No of respondents** | **No. of sisters survived (>15 years)** | **No. of sisters died from all maternal causes** | **No. of pregnancy-related deaths** | **Adjustment**  **factors** | **Sister unit of exposure (E)** |
| --- | --- | --- | --- | --- | --- | --- |
|  |  | **(A)** | **(B)** | **(C)** | **(D)** | **(E=A*D)** |
| 15-19 | 27 | 41* | 1 | 0 | 0.107 | 4 |
| 20-24 | 25 | 38* | 2 | 1 | 0.206 | 8 |
| 25-29 | 22 | 35 | 1 | 1 | 0.343 | 12 |
| 30-34 | 15 | 30 | 3 | 2 | 0.503 | 15 |
| 35-39 | 15 | 19 | 0 | 0 | 0.664 | 13 |
| 40-44 | 13 | 17 | 0 | 0 | 0.802 | 14 |
| 45-49 | 7 | 7 | 0 | 0 | 0.900 | 6 |
| **Total** | 124 | 187 | 7 | 4 |  | 72 |

* Adjusted number of sisters by multiplying the average number of sisters for respondents aged 25–49 (i.e., 1.50) by the number of respondents (age group 15–19 and 20–24). Originally the number of sisters was 32 for the age 15–19 years, and 37 for 20–24 years.

LTR = 4/72 = 0.056, TFR = 5.4 for 3-previous years, and MM Ratio = 1-(1- LTR)^1/TFR^ = 1062/100,000 LB (95%CI: 54, 2021).

**SNNP**

| **Age of respondents**  **in years** | **No of respondents** | **No. of sisters survived (>15 years)** | **No. of sisters died from all maternal causes** | **No. of pregnancy-related deaths** | **Adjustment**  **factors** | **Sister unit of exposure (E)** |
| --- | --- | --- | --- | --- | --- | --- |
|  |  | **(A)** | **(B)** | **(C)** | **(D)** | **(E=A*D)** |
| 15-19 | 652 | 1121* | 29 | 4 | 0.107 | 120 |
| 20-24 | 545 | 1091^ | 30 | 10 | 0.206 | 225 |
| 25-29 | 589 | 978 | 42 | 20 | 0.343 | 335 |
| 30-34 | 417 | 840 | 22 | 7 | 0.503 | 423 |
| 35-39 | 342 | 615 | 23 | 4 | 0.664 | 408 |
| 40-44 | 233 | 448 | 11 | 1 | 0.802 | 359 |
| 45-49 | 217 | 220 | 10 | 0 | 0.900 | 198 |
| **Total** | 2995 | 5313 | **167** | **46** |  | 2068 |

* Adjusted number of sisters by multiplying the average number of sisters for respondents aged 25–49 (i.e., 1.72) by the number of respondents (age group 15–19). Originally the number of sisters was 832 for 15–19 years.

^ No adjustment. As the number of sisters after adjustment was much less than the number of sisters reported, the actual number of sisters was used. Because, after adjustment, the number of sisters was 938 for the age 20–24 years.

LTR = 46/2068 = 0.022, TFR = 5.4 for 3-previous years, and MM Ratio = 1-(1- LTR)^1/TFR^ = 411/100,000 LB (95%CI: 288, 527).

**Gambella**

| **Age of respondents**  **in years** | **No of respondents** | **No. of sisters survived (>15 years)** | **No. of sisters died from all maternal causes** | **No. of pregnancy-related deaths** | **Adjustment**  **factors** | **Sister unit of exposure (E)** |
| --- | --- | --- | --- | --- | --- | --- |
|  |  | **(A)** | **(B)** | **(C)** | **(D)** | **(E=A*D)** |
| 15-19 | 8 | 10* | 0 | 0 | 0.107 | 1 |
| 20-24 | 9 | 13^ | 0 | 0 | 0.206 | 3 |
| 25-29 | 10 | 10 | 0 | 0 | 0.343 | 3 |
| 30-34 | 5 | 9 | 0 | 0 | 0.503 | 5 |
| 35-39 | 6 | 7 | 0 | 0 | 0.664 | 5 |
| 40-44 | 3 | 4 | 0 | 0 | 0.802 | 3 |
| 45-49 | 3 | 3 | 0 | 0 | 0.900 | 3 |
| **Total** | 44 | 56 | 0 | 0 |  | 22 |

* Adjusted number of sisters by multiplying the average number of sisters for respondents aged 25–49 (i.e., 1.22) by the number of respondents (age group 15–19). Originally the number of sisters was 9 for 15–19 years.

^ No adjustment. As the number of sisters after adjustment was much less than the number of sisters reported, the actual number of sisters was used. Because, after adjustment, the number of sisters was 11 for the age 20–24 years.

LTR = 0/22 = 0, TFR = 5.4 for 3-previous years, and MM Ratio = 1-(1- LTR)^1/TFR^ = 0/100,000 LB (95%CI: 0, 0).

**Harari**

| **Age of respondents**  **in years** | **No of respondents** | **No. of sisters survived (>15 years)** | **No. of sisters died from all maternal causes** | **No. of pregnancy-related deaths** | **Adjustment**  **Factors** | **Sister unit of exposure (E)** |
| --- | --- | --- | --- | --- | --- | --- |
|  |  | **(A)** | **(B)** | **(C)** | **(D)** | **(E=A*D)** |
| 15-19 | 11 | 21* | 0 | 0 | 0.107 | 2 |
| 20-24 | 7 | 13^ | 0 | 0 | 0.206 | 3 |
| 25-29 | 8 | 13 | 0 | 0 | 0.343 | 4 |
| 30-34 | 4 | 12 | 0 | 0 | 0.503 | 6 |
| 35-39 | 4 | 7 | 0 | 0 | 0.664 | 5 |
| 40-44 | 3 | 5 | 0 | 0 | 0.802 | 4 |
| 45-49 | 2 | 2 | 0 | 0 | 0.900 | 2 |
| **Total** | 39 | 73 | 0 | 0 |  | 26 |

* Adjusted number of sisters by multiplying the average number of sisters for respondents aged 25–49 (i.e., 1.86) by the number of respondents (age group 15–19). Originally the number of sisters was 10 for 15–19 years.

^ No adjustment. As the number of sisters after adjustment was almost equal to the number of sisters reported, the actual number of sisters was used. Because, after adjustment, the number of sisters was 13 for the age 20–24 years.

LTR = 0/26 = 0, TFR = 5.4 for 3-previous years, and MMRatio = 1-(1- LTR)^1/TFR^ = 0/100,000 LB (95%CI: 0, 0).

**Addis Ababa**

| **Age of respondents**  **in years** | **No of respondents** | **No. of sisters survived (>15 years)** | **No. of sisters died from all maternal causes** | **No. of pregnancy-related deaths** | **Adjustment**  **Factors** | **Sister unit of exposure (E)** |
| --- | --- | --- | --- | --- | --- | --- |
|  |  | **(A)** | **(B)** | **(C)** | **(D)** | **(E=A*D)** |
| 15-19 | 199 | 390* | 3 | 0 | 0.107 | 42 |
| 20-24 | 183 | 359* | 11 | 3 | 0.206 | 74 |
| 25-29 | 128 | 254 | 11 | 1 | 0.343 | 87 |
| 30-34 | 68 | 181 | 3 | 0 | 0.503 | 91 |
| 35-39 | 71 | 144 | 5 | 0 | 0.664 | 96 |
| 40-44 | 59 | 91 | 0 | 0 | 0.802 | 73 |
| 45-49 | 48 | 62 | 3 | 0 | 0.900 | 56 |
| **Total** | 756 | 1481 | **36** | **4** |  | 518 |

* Adjusted number of sisters by multiplying the average number of sisters for respondents aged 25–49 (i.e., 1.96) by the number of respondents (age group 15–19 and 20–24). Originally the number of sisters was 240 for the age 15–19 years, and 269 for 20–24 years.

LTR = 4/518 = 0.008, TFR = 5.4 for 3-previous years, and MM Ratio = 1-(1- LTR)^1/TFR^ = 149/100,000 LB (95%CI: 7, 290).

**Dire Dawa**

| **Age of respondents**  **in years** | **No of respondents** | **No. of sisters survived (>15 years)** | **No. of sisters died from all maternal causes** | **No. of pregnancy-related deaths** | **Adjustment**  **Factors** | **Sister unit of exposure (E)** |
| --- | --- | --- | --- | --- | --- | --- |
|  |  | **(A)** | **(B)** | **(C)** | **(D)** | **(E=A*D)** |
| 15-19 | 16 | 28* | 0 | 0 | 0.107 | 3 |
| 20-24 | 13 | 23* | 0 | 0 | 0.206 | 5 |
| 25-29 | 13 | 23 | 0 | 0 | 0.343 | 8 |
| 30-34 | 8 | 16 | 0 | 0 | 0.503 | 8 |
| 35-39 | 8 | 15 | 0 | 0 | 0.664 | 10 |
| 40-44 | 5 | 10 | 0 | 0 | 0.802 | 8 |
| 45-49 | 6 | 5 | 0 | 0 | 0.900 | 5 |
| **Total** | 69 | 120 | 0 | 0 |  | 46 |

* Adjusted number of sisters by multiplying the average number of sisters for respondents aged 25–49 (i.e., 1.73) by the number of respondents (age group 15–19 and 20–24). Originally the number of sisters was 18 for the age 15–19 years, and 22 for 20–24 years.

LTR = 0/46 = 0, TFR = 5.4 for 3-previous years, and MM Ratio = 1-(1- LTR)^1/TFR^ = 0/100,000 LB (95%CI: 0, 0).
