# Supplementary material for: A woman's lifetime risk disparities in maternal mortality in Ethiopia
Source: Public Health Chall. 2023 Jan 13;2(1):e56. doi: 10.1002/puh2.56 (PMC12039551; doi:10.1002/puh2.56)
Supplement: Supplementary file 3 — Estimation of lifetime risks of maternal mortality for the year 2011, Ethiopia [file PUH2-2-e56-s004.docx]

**Supplementary file 3.** Estimation of lifetime risks of maternal mortality for the year 2011, Ethiopia

**Place of residence**

**Urban**

| **Age of respondents**  **in years** | **No of respondents** | **No. of sisters survived**  **(>15 years)** | **No. of sisters died from all maternal causes** | **No. of pregnancy-related deaths** | **Adjustment**  **factors** | **Sister unit of exposure (E)** |
| --- | --- | --- | --- | --- | --- | --- |
|  |  | **(A)** | **(B)** | **(C)** | **(D)** | **(E=A*D)** |
| 15-19 | 1042 | 2115* | 19 | 6 | 0.107 | 226 |
| 20-24 | 836 | 1697* | 29 | 14 | 0.206 | 350 |
| 25-29 | 770 | 1511 | 23 | 7 | 0.343 | 518 |
| 30-34 | 482 | 1206 | 28 | 7 | 0.503 | 607 |
| 35-39 | 400 | 711 | 19 | 2 | 0.664 | 472 |
| 40-44 | 230 | 499 | 28 | 7 | 0.802 | 400 |
| 45-49 | 187 | 275 | 6 | 0 | 0.900 | 248 |
| **Total** | 3947 | 8014 | **152** | **43** |  | 2821 |

* Adjusted number of sisters by multiplying the average number of sisters for respondents aged 25–49 (i.e., 2.03) by the number of respondents (age group 15–19 and 20–24). Originally the number of sisters was 1175 for the age 15–19 years, and 1593 for 20–24 years.

LTR = 43/2821 = 0.015, TFR = 4.8 for 3-previous years, and MM Ratio = 1-(1- LTR)^1/TFR^ = 315/100,000 LB (95%CI: 219, 406).

**Rural**

| **Age of respondents**  **in years** | **No of respondents** | **No. of sisters survived (>15 years)** | **No. of sisters died from all maternal causes** | **No. of pregnancy-related deaths** | **Adjustment**  **factors** | **Sister unit of exposure (E)** |
| --- | --- | --- | --- | --- | --- | --- |
|  |  | **(A)** | **(B)** | **(C)** | **(D)** | **(E=A*D)** |
| 15-19 | 2968 | 5342* | 66 | 12 | 0.107 | 572 |
| 20-24 | 2095 | 4368^ | 73 | 23 | 0.206 | 900 |
| 25-29 | 2377 | 4120 | 98 | 30 | 0.343 | 1413 |
| 30-34 | 1572 | 3753 | 146 | 60 | 0.503 | 1888 |
| 35-39 | 1516 | 2717 | 56 | 26 | 0.664 | 1804 |
| 40-44 | 1031 | 1891 | 53 | 14 | 0.802 | 1517 |
| 45-49 | 1009 | 1040 | 40 | 5 | 0.900 | 936 |
| **Total** | 12568 | 23231 | **532** | **170** |  | 9029 |

* Adjusted number of sisters by multiplying the average number of sisters for respondents aged 25–49 (i.e., 1.80) by the number of respondents (age group 15–19). Originally the number of sisters was 3555 for 15–19 years.

^ No adjustment. As the number of sisters after adjustment was much less than the number of sisters reported, the actual number of sisters was used. Because, after adjustment, the number of sisters was 3771 for the age 20–24 years.

LTR = 170/9029 = 0.019, TFR = 4.8 for 3-previous years, and MM Ratio = 1-(1- LTR)^1/TFR^ = 399/100,000 LB (95%CI: 337, 455).

**Educational status**

**No education**

| **Age of respondents**  **in years** | **No of respondents** | **No. of sisters survived (>15 years)** | **No. of sisters died from all maternal causes** | **No. of pregnancy-related deaths** | **Adjustment**  **Factors** | **Sister unit of exposure (E)** |
| --- | --- | --- | --- | --- | --- | --- |
|  |  | **(A)** | **(B)** | **(C)** | **(D)** | **(E=A*D)** |
| 15-19 | 695 | 1956^ | 42 | 10 | 0.107 | 209 |
| 20-24 | 1114 | 2508^ | 61 | 20 | 0.206 | 517 |
| 25-29 | 1911 | 2630 | 60 | 15 | 0.343 | 902 |
| 30-34 | 1377 | 2820 | 111 | 49 | 0.503 | 1418 |
| 35-39 | 1292 | 2141 | 54 | 18 | 0.664 | 1422 |
| 40-44 | 988 | 1635 | 48 | 5 | 0.802 | 1311 |
| 45-49 | 1017 | 953 | 42 | 5 | 0.900 | 858 |
| **Total** | 8394 | 14643 | **418** | **122** |  | 6637 |

^ No adjustment. As the number of sisters after adjustment was much less than the number of sisters reported, the actual number of sisters was used. However, the adjustment factor for respondents aged 25–49 was 1.55. After adjustment, the number of sisters was 1077 for the age 15–19 years, and 1727 for 20–24 years.

LTR = 122/6637 = 0.019, TFR = 4.8 for 3-previous years, and MM Ratio = 1-(1- LTR)^1/TFR^ = 399/100,000 LB (95%CI: 327, 465).

**Primary**

| **Age of respondents**  **in years** | **No of respondents** | **No. of sisters survived (>15 years)** | **No. of sisters died from all maternal causes** | **No. of pregnancy-related deaths** | **Adjustment**  **Factors** | **Sister unit of exposure (E)** |
| --- | --- | --- | --- | --- | --- | --- |
|  |  | **(A)** | **(B)** | **(C)** | **(D)** | **(E=A*D)** |
| 15-19 | 2813 | 7005* | 39 | 9 | 0.107 | 750 |
| 20-24 | 1174 | 2923* | 37 | 18 | 0.206 | 602 |
| 25-29 | 923 | 2254 | 48 | 19 | 0.343 | 773 |
| 30-34 | 501 | 1594 | 50 | 17 | 0.503 | 802 |
| 35-39 | 501 | 985 | 20 | 12 | 0.664 | 654 |
| 40-44 | 211 | 585 | 21 | 11 | 0.802 | 469 |
| 45-49 | 152 | 279 | 6 | 0 | 0.900 | 251 |
| **Total** | 6275 | 15625 | **221** | **86** |  | 4301 |

* Adjusted number of sisters by multiplying the average number of sisters for respondents aged 25–49 (i.e., 2.49) by the number of respondents (age group 15–19 and 20–24). Originally the number of sisters was 2092 for the age 15–19 years, and 2671 for 20–24 years. LTR = 86/4301 = 0.020, TFR = 4.8 for 3-previous years, and MM Ratio = 1-(1- LTR)^1/TFR^ = 420/100,000 LB (95%CI: 327, 507).

**Secondary**

| **Age of respondents**  **in years** | **No of respondents** | **No. of sisters survived (>15 years)** | **No. of sisters died from all maternal causes** | **No. of pregnancy-related deaths** | **Adjustment**  **Factors** | **Sister unit of exposure (E)** |
| --- | --- | --- | --- | --- | --- | --- |
|  |  | **(A)** | **(B)** | **(C)** | **(D)** | **(E=A*D)** |
| 15-19 | 406 | 1186* | 1 | 0 | 0.107 | 127 |
| 20-24 | 345 | 1007* | 4 | 0 | 0.206 | 207 |
| 25-29 | 160 | 460 | 9 | 4 | 0.343 | 158 |
| 30-34 | 92 | 314 | 8 | 1 | 0.503 | 158 |
| 35-39 | 68 | 168 | 0 | 0 | 0.664 | 112 |
| 40-44 | 34 | 93 | 2 | 0 | 0.802 | 75 |
| 45-49 | 12 | 32 | 0 | 0 | 0.900 | 29 |
| **Total** | 1117 | 3260 | **24** | **5** |  | 865 |

* Adjusted number of sisters obtained by multiplying the average number of sisters for respondents aged 25–49 (i.e., 2.92) by the number of respondents (age group 15–19 and 20–24). Originally the number of sisters was 410 for the age 15–19 years, and 487 for 20–24 years. LTR = 5/865 = 0.006, TFR = 4.8 for 3-previous years, and MM Ratio = 1-(1- LTR)^1/TFR^ = 126/100,000 LB (95%CI: 19, 231).

**Higher**

| **Age of respondents**  **in years** | **No of respondents** | **No. of sisters survived (>15 years)** | **No. of sisters died from all maternal causes** | **No. of pregnancy-related deaths** | **Adjustment**  **Factors** | **Sister unit of exposure (E)** |
| --- | --- | --- | --- | --- | --- | --- |
|  |  | **(A)** | **(B)** | **(C)** | **(D)** | **(E=A*D)** |
| 15-19 | 95 | 272^ | 4 | 0 | 0.107 | 29 |
| 20-24 | 298 | 692* | 4 | 0 | 0.206 | 143 |
| 25-29 | 153 | 286 | 4 | 1 | 0.343 | 98 |
| 30-34 | 84 | 234 | 1 | 0 | 0.503 | 118 |
| 35-39 | 55 | 132 | 0 | 0 | 0.664 | 88 |
| 40-44 | 28 | 77 | 4 | 0 | 0.802 | 62 |
| 45-49 | 16 | 52 | 0 | 0 | 0.900 | 47 |
| **Total** | 729 | 1745 | **17** | **1** |  | 584 |

^ No adjustment. As the number of sisters after adjustment was much less than the number of sisters reported, the actual number of sisters was used. Because, after adjustment, the number of sisters was 220 for the age 15–19 years.

* Adjusted number of sisters by multiplying the average number of sisters for respondents aged 25–49 (i.e., 2.32) by the number of respondents (age group 20–24). Originally the number of sisters was 298 for 20–24 years.

LTR = 1/584 = 0.002, TFR = 4.8 for 3-previous years, and MM Ratio = 1-(1- LTR)^1/TFR^ = 42/100,000 LB (95%CI: -35, 120).

**Wealth quintile**

**Lowest**

| **Age of respondents**  **in years** | **No of respondents** | **No. of sisters survived (>15 years)** | **No. of sisters died from all maternal causes** | **No. of pregnancy-related deaths** | **Adjustment**  **factors** | **Sister unit of exposure (E)** |
| --- | --- | --- | --- | --- | --- | --- |
|  |  | **(A)** | **(B)** | **(C)** | **(D)** | **(E=A*D)** |
| 15-19 | 686 | 1187* | 13 | 2 | 0.107 | 127 |
| 20-24 | 430 | 956^ | 18 | 8 | 0.206 | 197 |
| 25-29 | 547 | 894 | 24 | 5 | 0.343 | 307 |
| 30-34 | 381 | 867 | 31 | 18 | 0.503 | 436 |
| 35-39 | 398 | 646 | 15 | 8 | 0.664 | 429 |
| 40-44 | 253 | 542 | 13 | 4 | 0.802 | 435 |
| 45-49 | 291 | 282 | 19 | 1 | 0.900 | 254 |
| **Total** | 2986 | 5374 | **133** | **46** |  | 2184 |

* Adjusted number of sisters by multiplying the average number of sisters for respondents aged 25–49 (i.e., 1.73) by the number of respondents (age group 15–19). Originally the number of sisters was 784 for 15–19 years.

^ No adjustment. As the number of sisters after adjustment was much less than the number of sisters reported, the actual number of sisters was used. Because, after adjustment, the number of sisters was 744 for the age 20–24 years.

LTR = 46/2184 = 0.021, TFR = 4.8 for 3-previous years, and MM Ratio = 1-(1- LTR)^1/TFR^ = 441/100,000 LB (95%CI: 311, 564).

**Second**

| **Age of respondents**  **in years** | **No of respondents** | **No. of sisters survived (>15 years)** | **No. of sisters died from all maternal causes** | **No. of pregnancy-related deaths** | **Adjustment**  **factors** | **Sister unit of exposure (E)** |
| --- | --- | --- | --- | --- | --- | --- |
|  |  | **(A)** | **(B)** | **(C)** | **(D)** | **(E=A*D)** |
| 15-19 | 696 | 1190* | 26 | 7 | 0.107 | 127 |
| 20-24 | 498 | 1069^ | 24 | 4 | 0.206 | 220 |
| 25-29 | 599 | 919 | 28 | 9 | 0.343 | 315 |
| 30-34 | 355 | 906 | 37 | 14 | 0.503 | 456 |
| 35-39 | 379 | 670 | 17 | 7 | 0.664 | 445 |
| 40-44 | 277 | 401 | 10 | 3 | 0.802 | 322 |
| 45-49 | 237 | 269 | 10 | 4 | 0.900 | 242 |
| **Total** | 3041 | 5424 | **152** | **48** |  | 2127 |

* Adjusted number of sisters by multiplying the average number of sisters for respondents aged 25–49 (i.e., 1.71) by the number of respondents (age group 15–19). Originally the number of sisters was 810 for 15–19 years.

^ No adjustment. As the number of sisters after adjustment was much less than the number of sisters reported, the actual number of sisters was used. Because, after adjustment, the number of sisters for the age 20–24 years, was 852.

LTR = 48/2127 = 0.023, TFR = 4.8 for 3-previous years, and MM Ratio= 1-(1- LTR)^1/TFR^ = 484/100,000 LB (95%CI: 345, 614).

**Middle**

| **Age of respondents**  **in years** | **No of respondents** | **No. of sisters survived (>15 years)** | **No. of sisters died from all maternal causes** | **No. of pregnancy-related deaths** | **Adjustment**  **factors** | **Sister unit of exposure (E)** |
| --- | --- | --- | --- | --- | --- | --- |
|  |  | **(A)** | **(B)** | **(C)** | **(D)** | **(E=A*D)** |
| 15-19 | 687 | 1237* | 21 | 4 | 0.107 | 132 |
| 20-24 | 503 | 1082^ | 13 | 2 | 0.206 | 223 |
| 25-29 | 582 | 1065 | 18 | 6 | 0.343 | 365 |
| 30-34 | 405 | 881 | 30 | 13 | 0.503 | 443 |
| 35-39 | 364 | 664 | 13 | 4 | 0.664 | 441 |
| 40-44 | 238 | 459 | 15 | 0 | 0.802 | 368 |
| 45-49 | 252 | 251 | 10 | 0 | 0.900 | 226 |
| **Total** | 3031 | 5639 | **120** | **29** |  | 2199 |

* Adjusted number of sisters by multiplying the average number of sisters for respondents aged 25–49 (i.e., 1.80) by the number of respondents (age group 15–19). Originally the number of sisters was 854 for 15–19 years.

^ No adjustment. As the number of sisters after adjustment was much less than the number of sisters reported, the actual number of sisters was used. Because, after adjustment, the number of sisters was 905 for the age 20–24 years.

LTR = 29/2199 = 0.013, TFR = 4.8 for 3-previous years, and MM Ratio = 1-(1- LTR)^1/TFR^ = 272/100,000 LB (95%CI: 172, 370).

**Fourth**

| **Age of respondents**  **in years** | **No of respondents** | **No. of sisters survived (>15 years)** | **No. of sisters died from all maternal causes** | **No. of pregnancy-related deaths** | **Adjustment**  **Factors** | **Sister unit of exposure (E)** |
| --- | --- | --- | --- | --- | --- | --- |
|  |  | **(A)** | **(B)** | **(C)** | **(D)** | **(E=A*D)** |
| 15-19 | 889 | 1734* | 8 | 0 | 0.107 | 186 |
| 20-24 | 527 | 1149^ | 24 | 10 | 0.206 | 237 |
| 25-29 | 591 | 1127 | 25 | 6 | 0.343 | 387 |
| 30-34 | 379 | 998 | 40 | 15 | 0.503 | 502 |
| 35-39 | 346 | 693 | 18 | 6 | 0.664 | 460 |
| 40-44 | 253 | 460 | 17 | 1 | 0.802 | 369 |
| 45-49 | 230 | 232 | 5 | 0 | 0.900 | 209 |
| **Total** | 3215 | 6393 | **137** | **38** |  | 2349 |

* Adjusted number of sisters by multiplying the average number of sisters for respondents aged 25–49 (i.e., 1.95) by the number of respondents (age group 15–19). Originally the number of sisters was 959 for 15–19 years.

^ No adjustment. As the number of sisters after adjustment was much less than the number of sisters reported, the actual number of sisters was used. Because, after adjustment, the number of sisters was 1028 for the age 20–24 years.

LTR = 38/2349 = 0.016, TFR = 4.8 for 3-previous years, and MM Ratio = 1-(1- LTR)^1/TFR^ = 336/100,000 LB (95%CI: 227, 440).

**Highest**

| **Age of respondents**  **in years** | **No of respondents** | **No. of sisters survived (>15 years)** | **No. of sisters died from all maternal causes** | **No. of pregnancy-related deaths** | **Adjustment**  **factors** | **Sister unit of exposure (E)** |
| --- | --- | --- | --- | --- | --- | --- |
|  |  | **(A)** | **(B)** | **(C)** | **(D)** | **(E=A*D)** |
| 15-19 | 1051 | 2134* | 20 | 6 | 0.107 | 228 |
| 20-24 | 973 | 1975* | 26 | 13 | 0.206 | 407 |
| 25-29 | 827 | 1627 | 29 | 10 | 0.343 | 558 |
| 30-34 | 535 | 1308 | 38 | 8 | 0.503 | 658 |
| 35-39 | 430 | 757 | 15 | 4 | 0.664 | 503 |
| 40-44 | 240 | 525 | 29 | 7 | 0.802 | 421 |
| 45-49 | 187 | 282 | 5 | 0 | 0.900 | 254 |
| **Total** | 4243 | 8608 | **162** | **48** |  | 3029 |

* Adjusted number of sisters by multiplying the average number of sisters for respondents aged 25–49 (i.e., 2.03) by the number of respondents (age group 15–19 and 20–24). Originally the number of sisters was 1324 for the age 15–19 years, and 1707 for 20–24 years. LTR = 48/3029 = 0.016, TFR = 4.8 for 3-previous years, and MM Ratio = 1-(1- LTR)^1/TFR^ = 336/100,000 LB (95%CI:240, 427).

**Sub-national administrative regions**

**Tigray**

| **Age of respondents**  **in years** | **No of respondents** | **No. of sisters survived (>15 years)** | **No. of sisters died from all maternal causes** | **No. of pregnancy-related deaths** | **Adjustment**  **factors** | **Sister unit of exposure (E)** |
| --- | --- | --- | --- | --- | --- | --- |
|  |  | **(A)** | **(B)** | **(C)** | **(D)** | **(E=A*D)** |
| 15-19 | 294 | 521* | 7 | 3 | 0.107 | 56 |
| 20-24 | 200 | 357^ | 7 | 3 | 0.206 | 74 |
| 25-29 | 171 | 320 | 14 | 3 | 0.343 | 110 |
| 30-34 | 119 | 298 | 15 | 4 | 0.503 | 150 |
| 35-39 | 143 | 210 | 4 | 2 | 0.664 | 139 |
| 40-44 | 95 | 155 | 5 | 1 | 0.802 | 124 |
| 45-49 | 81 | 94 | 5 | 1 | 0.900 | 85 |
| **Total** | 1103 | 1955 | **57** | **17** |  | 737 |

* Adjusted number of sisters by multiplying the average number of sisters for respondents aged 25–49 (i.e., 1.77) by the number of respondents (age group 15–19). Originally the number of sisters was 279 for 15–19 years.

^ No adjustment. As the number of sisters after adjustment was much less than the number of sisters reported, the actual number of sisters was used. Because, after adjustment, the number of sisters was 354 for the age 20–24 years.

LTR = 17/737 = 0.023, TFR = 4.8 for 3-previous years, and MM Ratio = 1-(1- LTR)^1/TFR^ = 484/100,000 LB (95%CI: 254, 705).

**Afar**

| **Age of respondents**  **in years** | **No of respondents** | **No. of sisters survived (>15 years)** | **No. of sisters died from all maternal causes** | **No. of pregnancy-related deaths** | **Adjustment**  **factors** | **Sister unit of exposure (E)** |
| --- | --- | --- | --- | --- | --- | --- |
|  |  | **(A)** | **(B)** | **(C)** | **(D)** | **(E=A*D)** |
| 15-19 | 30 | 45* | 0 | 0 | 0.107 | 5 |
| 20-24 | 28 | 52^ | 0 | 0 | 0.206 | 11 |
| 25-29 | 31 | 46 | 0 | 0 | 0.343 | 16 |
| 30-34 | 16 | 35 | 0 | 0 | 0.503 | 18 |
| 35-39 | 17 | 22 | 0 | 0 | 0.664 | 15 |
| 40-44 | 13 | 16 | 0 | 0 | 0.802 | 13 |
| 45-49 | 9 | 9 | 0 | 0 | 0.900 | 8 |
| **Total** | 144 | 225 | **0** | **0** |  | 84 |

* Adjusted number of sisters by multiplying the average number of sisters for respondents aged 25–49 (i.e., 1.49) by the number of respondents (age group 15–19). Originally the number of sisters was 39 for 15–19 years.

^ No adjustment. As the number of sisters after adjustment was much less than the number of sisters reported, the actual number of sisters was used. Because, after adjustment, the number of sisters was 42 for the age 20–24 years.

LTR = 0/84 = 0, TFR = 4.8 for 3-previous years, and MM Ratio = 1-(1- LTR)^1/TFR^ = 0/100,000 LB (95%CI: 0, 0).

**Amhara**

| **Age of respondents**  **in years** | **No of respondents** | **No. of sisters survived (>15 years)** | **No. of sisters died from all maternal causes** | **No. of pregnancy-related deaths** | **Adjustment**  **factors** | **Sister unit of exposure (E)** |
| --- | --- | --- | --- | --- | --- | --- |
|  |  | **(A)** | **(B)** | **(C)** | **(D)** | **(E=A*D)** |
| 15-19 | 1123 | 1965* | 26 | 0 | 0.107 | 210 |
| 20-24 | 766 | 1541^ | 26 | 6 | 0.206 | 317 |
| 25-29 | 728 | 1417 | 20 | 8 | 0.343 | 486 |
| 30-34 | 541 | 1207 | 40 | 9 | 0.503 | 607 |
| 35-39 | 541 | 849 | 22 | 10 | 0.664 | 564 |
| 40-44 | 349 | 613 | 28 | 7 | 0.802 | 492 |
| 45-49 | 387 | 374 | 12 | 2 | 0.900 | 337 |
| **Total** | 4435 | 7966 | **174** | **42** |  | 3013 |

* Adjusted number of sisters by multiplying the average number of sisters for respondents aged 25–49 (i.e., 1.75) by the number of respondents (age group 15–19). Originally the number of sisters was 1186 for 15–19 years.

^ No adjustment. As the number of sisters after adjustment was much less than the number of sisters reported, the actual number of sisters was used. Because, after adjustment, the number of sisters was 1341 for the age 20–24 years.

LTR = 42/3013 = 0.014, TFR = 4.8 for 3-previous years, and MM Ratio = 1-(1- LTR)^1/TFR^ = 293/100,000 LB (95%CI: 204, 379).

**Oromiya**

| **Age of respondents**  **in years** | **No of respondents** | **No. of sisters survived (>15 years)** | **No. of sisters died from all maternal causes** | **No. of pregnancy-related deaths** | **Adjustment**  **factors** | **Sister unit of exposure (E)** |
| --- | --- | --- | --- | --- | --- | --- |
|  |  | **(A)** | **(B)** | **(C)** | **(D)** | **(E=A*D)** |
| 15-19 | 1489 | 2844* | 15 | 9 | 0.107 | 304 |
| 20-24 | 1043 | 2264^ | 45 | 16 | 0.206 | 466 |
| 25-29 | 1274 | 2130 | 43 | 7 | 0.343 | 731 |
| 30-34 | 720 | 1888 | 79 | 41 | 0.503 | 950 |
| 35-39 | 641 | 1278 | 17 | 3 | 0.664 | 849 |
| 40-44 | 431 | 899 | 32 | 6 | 0.802 | 721 |
| 45-49 | 412 | 460 | 23 | 0 | 0.900 | 414 |
| **Total** | 6010 | 11763 | **254** | **82** |  | 4435 |

* Adjusted number of sisters by multiplying the average number of sisters for respondents aged 25–49 (i.e., 1.91) by the number of respondents (age group 15–19). Originally the number of sisters was 1935 for 15–19 years.

^ No adjustment. As the number of sisters after adjustment was much less than the number of sisters reported, the actual number of sisters was used. Because, after adjustment, the number of sisters was 1992 for the age 20–24 years.

LTR = 82/4435 = 0.019, TFR = 4.8 for 3-previous years, and MM Ratio = 1-(1- LTR)^1/TFR^ = 399/100,000 LB (95%CI: 311, 482).

**Somali**

| **Age of respondents**  **in years** | **No of respondents** | **No. of sisters survived (>15 years)** | **No. of sisters died from all maternal causes** | **No. of pregnancy-related deaths** | **Adjustment**  **factors** | **Sister unit of exposure (E)** |
| --- | --- | --- | --- | --- | --- | --- |
|  |  | **(A)** | **(B)** | **(C)** | **(D)** | **(E=A*D)** |
| 15-19 | 70 | 123* | 4 | 2 | 0.107 | 13 |
| 20-24 | 51 | 124^ | 5 | 1 | 0.206 | 26 |
| 25-29 | 65 | 109 | 0 | 0 | 0.343 | 37 |
| 30-34 | 48 | 104 | 2 | 1 | 0.503 | 52 |
| 35-39 | 46 | 79 | 0 | 0 | 0.664 | 52 |
| 40-44 | 32 | 47 | 1 | 0 | 0.802 | 38 |
| 45-49 | 17 | 24 | 1 | 0 | 0.900 | 22 |
| **Total** | 329 | 610 | **13** | **4** |  | 240 |

* Adjusted number of sisters by multiplying the average number of sisters for respondents aged 25–49 (i.e., 1.75) by the number of respondents (age group 15–19). Originally the number of sisters was 105 for 15–19 years.

^ No adjustment. As the number of sisters after adjustment was much less than the number of sisters reported, the actual number of sisters was used. Because, after adjustment, the number of sisters was 89 for the age 20–24 years.

LTR = 4/240 = 0.017, TFR = 4.8 for 3-previous years, and MM Ratio = 1-(1- LTR)^1/TFR^ = 357/100,000 LB (95%CI: 11, 697).

**Beshngul Gumuz**

| **Age of respondents**  **in years** | **No of respondents** | **No. of sisters survived (>15 years)** | **No. of sisters died from all maternal causes** | **No. of pregnancy-related deaths** | **Adjustment**  **factors** | **Sister unit of exposure (E)** |
| --- | --- | --- | --- | --- | --- | --- |
|  |  | **(A)** | **(B)** | **(C)** | **(D)** | **(E=A*D)** |
| 15-19 | 39 | 65* | 0 | 0 | 0.107 | 7 |
| 20-24 | 33 | 56^ | 0 | 0 | 0.206 | 12 |
| 25-29 | 36 | 56 | 0 | 0 | 0.343 | 19 |
| 30-34 | 22 | 46 | 0 | 0 | 0.503 | 23 |
| 35-39 | 20 | 31 | 0 | 0 | 0.664 | 21 |
| 40-44 | 14 | 20 | 0 | 0 | 0.802 | 16 |
| 45-49 | 10 | 10 | 0 | 0 | 0.900 | 9 |
| **Total** | 174 | 284 | 0 | 0 |  | 106 |

* Adjusted number of sisters by multiplying the average number of sisters for respondents aged 25–49 (i.e., 1.60) by the number of respondents (age group 15–19). Originally the number of sisters was 42 for 15–19 years.

^ No adjustment. As the number of sisters after adjustment was much less than the number of sisters reported, the actual number of sisters was used. Because, after adjustment, the number of sisters was 53 for the age 20–24 years.

LTR = 0/106 = 0, TFR = 4.8 for 3-previous years, and MM Ratio = 1-(1- LTR)^1/TFR^ = 0/100,000 LB (95%CI: 0, 0).

**SNNP**

| **Age of respondents**  **in years** | **No of respondents** | **No. of sisters survived (>15 years)** | **No. of sisters died from all maternal causes** | **No. of pregnancy-related deaths** | **Adjustment**  **factors** | **Sister unit of exposure (E)** |
| --- | --- | --- | --- | --- | --- | --- |
|  |  | **(A)** | **(B)** | **(C)** | **(D)** | **(E=A*D)** |
| 15-19 | 710 | 1363* | 28 | 3 | 0.107 | 146 |
| 20-24 | 566 | 1182^ | 19 | 5 | 0.206 | 243 |
| 25-29 | 613 | 1141 | 34 | 15 | 0.343 | 391 |
| 30-34 | 446 | 1069 | 26 | 8 | 0.503 | 538 |
| 35-39 | 400 | 758 | 26 | 13 | 0.664 | 503 |
| 40-44 | 270 | 522 | 8 | 2 | 0.802 | 419 |
| 45-49 | 231 | 274 | 4 | 0 | 0.900 | 247 |
| **Total** | 3236 | 6309 | **145** | **46** |  | 2487 |

* Adjusted number of sisters by multiplying the average number of sisters for respondents aged 25–49 (i.e., 1.92) by the number of respondents (age group 15–19). Originally the number of sisters was 827 for 15–19 years.

^ No adjustment. As the number of sisters after adjustment was much less than the number of sisters reported, the actual number of sisters was used. Because, after adjustment, the number of sisters was 1087 for the age 20–24 years.

LTR = 46/2487 = 0.019, TFR = 4.8 for 3-previous years, and MM Ratio = 1-(1- LTR)^1/TFR^ = 399/100,000 LB (95%CI: 284, 508).

**Gambella**

| **Age of respondents**  **in years** | **No of respondents** | **No. of sisters survived (>15 years)** | **No. of sisters died from all maternal causes** | **No. of pregnancy-related deaths** | **Adjustment**  **factors** | **Sister unit of exposure (E)** |
| --- | --- | --- | --- | --- | --- | --- |
|  |  | **(A)** | **(B)** | **(C)** | **(D)** | **(E=A*D)** |
| 15-19 | 18 | 29* | 0 | 0 | 0.107 | 3 |
| 20-24 | 17 | 27* | 0 | 0 | 0.206 | 6 |
| 25-29 | 13 | 21 | 0 | 0 | 0.343 | 7 |
| 30-34 | 8 | 16 | 0 | 0 | 0.503 | 8 |
| 35-39 | 6 | 9 | 0 | 0 | 0.664 | 6 |
| 40-44 | 4 | 6 | 0 | 0 | 0.802 | 5 |
| 45-49 | 3 | 2 | 0 | 0 | 0.900 | 2 |
| **Total** | 69 | 110 | 0 | 0 |  | 37 |

* Adjusted number of sisters by multiplying the average number of sisters for respondents aged 25–49 (i.e., 1.59) by the number of respondents (age group 15–19 and 20–24). Originally the number of sisters was 17 for the age 15–19 years, and 19 for 20–24 years.

LTR = 0/37 = 0, TFR = 4.8 for 3-previous years, and MM Ratio = 1-(1- LTR)^1/TFR^ = 0/100,000 LB (95%CI: 0, 0).

**Harari**

| **Age of respondents**  **in years** | **No of respondents** | **No. of sisters survived (>15 years)** | **No. of sisters died from all maternal causes** | **No. of pregnancy-related deaths** | **Adjustment**  **Factors** | **Sister unit of exposure (E)** |
| --- | --- | --- | --- | --- | --- | --- |
|  |  | **(A)** | **(B)** | **(C)** | **(D)** | **(E=A*D)** |
| 15-19 | 11 | 20* | 0 | 0 | 0.107 | 2 |
| 20-24 | 10 | 18^ | 0 | 0 | 0.206 | 4 |
| 25-29 | 10 | 19 | 0 | 0 | 0.343 | 7 |
| 30-34 | 6 | 14 | 0 | 0 | 0.503 | 7 |
| 35-39 | 6 | 9 | 0 | 0 | 0.664 | 6 |
| 40-44 | 3 | 5 | 0 | 0 | 0.802 | 4 |
| 45-49 | 3 | 3 | 0 | 0 | 0.900 | 3 |
| **Total** | 49 | 88 | 0 | 0 |  | 32 |

* Adjusted number of sisters by multiplying the average number of sisters for respondents aged 25–49 (i.e., 1.79) by the number of respondents (age group 15–19). Originally the number of sisters was 14 for 15–19 years.

^ No adjustment. As the number of sisters after adjustment was equal to the number of sisters reported, the actual number of sisters was used. Because, after adjustment, the number of sisters was 18 for the age 20–24 years.

LTR = 0/32 = 0, TFR = 4.8 for 3-previous years, and MM Ratio = 1-(1- LTR)^1/TFR^ = 0/100,000 LB (95%CI: 0, 0).

**Addis Ababa**

| **Age of respondents**  **in years** | **No of respondents** | **No. of sisters survived (>15 years)** | **No. of sisters died from all maternal causes** | **No. of pregnancy-related deaths** | **Adjustment**  **Factors** | **Sister unit of exposure (E)** |
| --- | --- | --- | --- | --- | --- | --- |
|  |  | **(A)** | **(B)** | **(C)** | **(D)** | **(E=A*D)** |
| 15-19 | 210 | 405* | 7 | 1 | 0.107 | 43 |
| 20-24 | 203 | 392* | 6 | 2 | 0.206 | 81 |
| 25-29 | 191 | 344 | 10 | 2 | 0.343 | 118 |
| 30-34 | 120 | 264 | 11 | 3 | 0.503 | 133 |
| 35-39 | 89 | 166 | 2 | 1 | 0.664 | 110 |
| 40-44 | 45 | 98 | 6 | 0 | 0.802 | 79 |
| 45-49 | 39 | 61 | 3 | 0 | 0.900 | 55 |
| **Total** | 897 | 1730 | **45** | **9** |  | 619 |

* Adjusted number of sisters by multiplying the average number of sisters for respondents aged 25–49 (i.e., 1.93) by the number of respondents (age group 15–19 and 20–24). Originally the number of sisters was 269 for the age 15–19 years, and 325 for 20–24 years.

LTR = 9/619 = 0.015, TFR = 4.8 for 3-previous years, and MM Ratio = 1-(1- LTR)^1/TFR^ = 315/100,000 LB (95%CI: 113, 512).

**Dire Dawa**

| **Age of respondents**  **in years** | **No of respondents** | **No. of sisters survived (>15 years)** | **No. of sisters died from all maternal causes** | **No. of pregnancy-related deaths** | **Adjustment**  **Factors** | **Sister unit of exposure (E)** |
| --- | --- | --- | --- | --- | --- | --- |
|  |  | **(A)** | **(B)** | **(C)** | **(D)** | **(E=A*D)** |
| 15-19 | 15 | 24* | 0 | 0 | 0.107 | 3 |
| 20-24 | 13 | 25^ | 0 | 0 | 0.206 | 5 |
| 25-29 | 15 | 24 | 0 | 0 | 0.343 | 8 |
| 30-34 | 9 | 18 | 0 | 0 | 0.503 | 9 |
| 35-39 | 9 | 11 | 0 | 0 | 0.664 | 7 |
| 40-44 | 5 | 9 | 0 | 0 | 0.802 | 7 |
| 45-49 | 3 | 4 | 0 | 0 | 0.900 | 4 |
| **Total** | 69 | 115 | 0 | 0 |  | 43 |

* Adjusted number of sisters by multiplying the average number of sisters for respondents aged 25–49 (i.e., 1.61) by the number of respondents (age group 15–19). Originally the number of sisters was 17 for 15–19 years.

^ No adjustment. As the number of sisters after adjustment was much less than the number of sisters reported, the actual number of sisters was used. Because, after adjustment, the number of sisters was 21 for the age 20–24 years.

LTR = 0/43 = 0, TFR = 4.8 for 3-prevoius years, and MM Ratio = 1-(1- LTR)^1/TFR^ = 0/100,000 LB (95%CI: 0, 0).
