# Supplementary material for: A woman's lifetime risk disparities in maternal mortality in Ethiopia
Source: Public Health Chall. 2023 Jan 13;2(1):e56. doi: 10.1002/puh2.56 (PMC12039551; doi:10.1002/puh2.56)
Supplement: Supplementary file 4 — Estimation of lifetime risks of maternal mortality for the year 2016, Ethiopia [file PUH2-2-e56-s002.docx]

**Supplementary file 4.** Estimation of lifetime risks of maternal mortality for the year 2016, Ethiopia

**Place of residence**

**Urban**

| **Age of respondents**  **in years** | **No of respondents** | **No. of sisters survived**  **(>15 years)** | **No. of sisters died from all maternal causes** | **No. of pregnancy-related deaths** | **Adjustment**  **factors** | **Sister unit of exposure (E)** |
| --- | --- | --- | --- | --- | --- | --- |
|  |  | **(A)** | **(B)** | **(C)** | **(D)** | **(E=A*D)** |
| 15-19 | 805 | 1554* | 20 | 0 | 0.107 | 166 |
| 20-24 | 663 | 1280* | 15 | 8 | 0.206 | 264 |
| 25-29 | 732 | 1222 | 7 | 1 | 0.343 | 419 |
| 30-34 | 486 | 1058 | 28 | 7 | 0.503 | 532 |
| 35-39 | 387 | 826 | 16 | 2 | 0.664 | 548 |
| 40-44 | 220 | 506 | 11 | 0 | 0.802 | 406 |
| 45-49 | 183 | 257 | 6 | 0 | 0.900 | 231 |
| **Total** | 3476 | **6702** | **103** | **18** |  | 2567 |

* Adjusted number of sisters by multiplying the average number of sisters for respondents aged 25–49 (i.e., 1.93) by the number of respondents (age group 15–19 and 20–24). Originally the number of sisters was 895 for the age 15–19 years, and 1163 for 20–24 years.

LTR = 18/2567 = 0.007, TFR = 4.6 for 3-previous years, and MM Ratio = 1-(1- LTR)^1/TFR^ = 153/100,000 LB (95%CI: 80, 224).

**Rural**

| **Age of respondents**  **in years** | **No of respondents** | **No. of sisters survived (>15 years)** | **No. of sisters died from all maternal causes** | **No. of pregnancy-related deaths** | **Adjustment**  **factors** | **Sister unit of exposure (E)** |
| --- | --- | --- | --- | --- | --- | --- |
|  |  | **(A)** | **(B)** | **(C)** | **(D)** | **(E=A*D)** |
| 15-19 | 2576 | 5768* | 42 | 8 | 0.107 | 617 |
| 20-24 | 2099 | 7216* | 52 | 17 | 0.206 | 1487 |
| 25-29 | 2224 | 4112 | 62 | 20 | 0.343 | 1410 |
| 30-34 | 1859 | 3661 | 57 | 20 | 0.503 | 1841 |
| 35-39 | 1545 | 2934 | 24 | 5 | 0.664 | 1948 |
| 40-44 | 1070 | 1857 | 29 | 7 | 0.802 | 1489 |
| 45-49 | 833 | 1126 | 17 | 5 | 0.900 | 1013 |
| **Total** | 12206 | **26674** | **283** | **82** |  | 9806 |

* Adjusted number of sisters by multiplying the average number of sisters for respondents aged 25–49 (i.e., 1.82) by the number of respondents (age group 15–19 and 20–24). Originally the number of sisters was 3169 for the age 15–19 years, and 3965 for 20–24 years. LTR = 82/9806 = 0.009, TFR = 4.6 for 3-previous years, and MM Ratio = 1-(1- LTR)^1/TFR^ = 196/100,000 LB (95%CI: 154, 237).

**Educational status**

**No education**

| **Age of respondents**  **in years** | **No of respondents** | **No. of sisters survived (>15 years)** | **No. of sisters died from all maternal causes** | **No. of pregnancy-related deaths** | **Adjustment**  **factors** | **Sister unit of exposure (E)** |
| --- | --- | --- | --- | --- | --- | --- |
|  |  | **(A)** | **(B)** | **(C)** | **(D)** | **(E=A*D)** |
| 15-19 | 469 | 1452^ | 26 | 2 | 0.107 | 155 |
| 20-24 | 762 | 2101^ | 37 | 7 | 0.206 | 433 |
| 25-29 | 1493 | 2534 | 48 | 19 | 0.343 | 869 |
| 30-34 | 1665 | 2510 | 54 | 16 | 0.503 | 1263 |
| 35-39 | 1371 | 2237 | 22 | 8 | 0.664 | 1485 |
| 40-44 | 940 | 1489 | 27 | 8 | 0.802 | 1194 |
| 45-49 | 798 | 907 | 17 | 5 | 0.900 | 816 |
| **Total** | **7498** | **13230** | **231** | **65** |  | **6216** |

^ No adjustment. As the number of sisters after adjustment was much less than the number of sisters reported, the actual number of sisters was used. However, the adjustment factor for respondents aged 25–49 was 1.54. After adjustment, the number of sisters was 722 for the age 15–19 years, and 1174 for 20–24 years.

LTR = 65/6216 = 0.011, TFR = 4.6 for 3-previous years, and MM Ratio = 1-(1- LTR)^1/TFR^ = 240/100,000 LB (95%CI: 185, 296).

**Primary**

| **Age of respondents**  **in years** | **No of respondents** | **No. of sisters survived (>15 years)** | **No. of sisters died from all maternal causes** | **No. of pregnancy-related deaths** | **Adjustment**  **factors** | **Sister unit of exposure (E)** |
| --- | --- | --- | --- | --- | --- | --- |
|  |  | **(A)** | **(B)** | **(C)** | **(D)** | **(E=A*D)** |
| 15-19 | 2148 | 5069* | 34 | 6 | 0.107 | 542 |
| 20-24 | 1185 | 2797* | 29 | 6 | 0.206 | 576 |
| 25-29 | 888 | 1820 | 27 | 4 | 0.343 | 624 |
| 30-34 | 468 | 1399 | 29 | 11 | 0.503 | 704 |
| 35-39 | 391 | 961 | 14 | 2 | 0.664 | 638 |
| 40-44 | 251 | 583 | 15 | 1 | 0.802 | 468 |
| 45-49 | 160 | 321 | 5 | 0 | 0.900 | 289 |
| **Total** | **5491** | **12950** | **153** | **30** |  | **3841** |

* Adjusted number of sisters by multiplying the average number of sisters for respondents aged 25–49 (i.e, 2.36) by the number of respondents (age group 15–19 and 20–24). Originally the number of sisters was 1715 for the age 15–19 years, and 2059 for 20–24 years. LTR = 30/3841 = 0.008, TFR = 4.6 for 3-previous years, and MM Ratio = 1-(1- LTR)^1/TFR^ = 175/100,000 LB (95%CI: 111, 237).

**Secondary**

| **Age of respondents**  **in years** | **No of respondents** | **No. of sisters survived (>15 years)** | **No. of sisters died from all maternal causes** | **No. of pregnancy-related deaths** | **Adjustment**  **Factors** | **Sister unit of exposure (E)** |
| --- | --- | --- | --- | --- | --- | --- |
|  |  | **(A)** | **(B)** | **(C)** | **(D)** | **(E=A*D)** |
| 15-19 | 678 | 1926* | 13 | 2 | 0.107 | 206 |
| 20-24 | 506 | 1437* | 20 | 12 | 0.206 | 296 |
| 25-29 | 326 | 659 | 3 | 0 | 0.343 | 226 |
| 30-34 | 107 | 519 | 8 | 3 | 0.503 | 261 |
| 35-39 | 106 | 337 | 4 | 0 | 0.664 | 224 |
| 40-44 | 61 | 182 | 1 | 0 | 0.802 | 146 |
| 45-49 | 33 | 98 | 5 | 0 | 0.900 | 88 |
| **Total** | **1817** | **5158** | **54** | **17** |  | **1447** |

* Adjusted number of sisters obtained by multiplying the average number of sisters for respondents aged 25–49 (i.e., 2.84) by the number of respondents (age group 15–19 and 20–24). Originally the number of sisters was 622 for the age 15–19 years, and 668 for 20–24 years. LTR = 17/1447 = 0.012, TFR = 4.6 for 3-previous years, and MM Ratio = 1-(1- LTR)^1/TFR^ = 262/100,000 LB (95%CI: 137, 385).

**Higher**

| **Age of respondents**  **in years** | **No of respondents** | **No. of sisters survived (>15 years)** | **No. of sisters died from all maternal causes** | **No. of pregnancy-related deaths** | **Adjustment**  **Factors** | **Sister unit of exposure (E)** |
| --- | --- | --- | --- | --- | --- | --- |
|  |  | **(A)** | **(B)** | **(C)** | **(D)** | **(E=A*D)** |
| 15-19 | 87 | 273^ | 1 | 0 | 0.107 | 29 |
| 20-24 | 309 | 643* | 1 | 0 | 0.206 | 132 |
| 25-29 | 250 | 320 | 0 | 0 | 0.343 | 110 |
| 30-34 | 104 | 289 | 2 | 1 | 0.503 | 145 |
| 35-39 | 64 | 222 | 0 | 0 | 0.664 | 147 |
| 40-44 | 37 | 109 | 0 | 0 | 0.802 | 87 |
| 45-49 | 25 | 59 | 1 | 0 | 0.900 | 53 |
| **Total** | **876** | **1915** | **5** | **1** |  | **705** |

^ No adjustment. As the number of sisters after adjustment was much less than the number of sisters reported, the actual number of sisters was used. Because, after adjustment, the number of sisters was 181 for the age 15–19 years.

* Adjusted number of sisters by multiplying the average number of sisters for respondents aged 25–49 (i.e., 2.08) by the number of respondents (age group 20–24). Originally the number of sisters was 299 for 20–24 years.

LTR = 1/705 = 0.002, TFR = 4.6 for 3-previous years, and MM Ratio = 1-(1- LTR)^1/TFR^ = 44/100,000 LB (95%CI: -28, 115).

**Wealth quintile**

**Lowest**

| **Age of respondents**  **in years** | **No of respondents** | **No. of sisters survived (>15 years)** | **No. of sisters died from all maternal causes** | **No. of pregnancy-related deaths** | **Adjustment**  **factors** | **Sister unit of exposure (E)** |
| --- | --- | --- | --- | --- | --- | --- |
|  |  | **(A)** | **(B)** | **(C)** | **(D)** | **(E=A*D)** |
| 15-19 | 478 | 794* | 15 | 4 | 0.107 | 85 |
| 20-24 | 468 | 819^ | 16 | 4 | 0.206 | 169 |
| 25-29 | 486 | 870 | 15 | 3 | 0.343 | 298 |
| 30-34 | 433 | 730 | 22 | 4 | 0.503 | 367 |
| 35-39 | 362 | 623 | 8 | 2 | 0.664 | 414 |
| 40-44 | 250 | 354 | 8 | 5 | 0.802 | 284 |
| 45-49 | 156 | 223 | 2 | 0 | 0.900 | 201 |
| **Total** | **2633** | **4413** | **86** | **22** |  | **1818** |

* Adjusted number of sisters by multiplying the average number of sisters for respondents aged 25–49 (i.e., 1.66) by the number of respondents (age group 15–19). Originally the number of sisters was 680 for 15–19 years.

^ No adjustment. As the number of sisters after adjustment was much less than the number of sisters reported, the actual number of sisters was used. Because, after adjustment, the number of sisters was 777 for the age 20–24 years.

LTR = 22/1818 = 0.012, TFR = 4.6 for 3-previous years, and MM Ratio = 1-(1- LTR)^1/TFR^ = 262/100,000 LB (95%CI: 35, 372).

**Second**

| **Age of respondents**  **in years** | **No of respondents** | **No. of sisters survived (>15 years)** | **No. of sisters died from all maternal causes** | **No. of pregnancy-related deaths** | **Adjustment**  **factors** | **Sister unit of exposure (E)** |
| --- | --- | --- | --- | --- | --- | --- |
|  |  | **(A)** | **(B)** | **(C)** | **(D)** | **(E=A*D)** |
| 15-19 | 558 | 927* | 14 | 0 | 0.107 | 99 |
| 20-24 | 523 | 868* | 23 | 4 | 0.206 | 179 |
| 25-29 | 564 | 865 | 22 | 5 | 0.343 | 297 |
| 30-34 | 417 | 782 | 14 | 4 | 0.503 | 393 |
| 35-39 | 325 | 590 | 6 | 0 | 0.664 | 392 |
| 40-44 | 228 | 402 | 1 | 0 | 0.802 | 322 |
| 45-49 | 195 | 228 | 9 | 2 | 0.900 | 205 |
| **Total** | **2810** | **4662** | **89** | **15** |  | **1887** |

* Adjusted number of sisters by multiplying the average number of sisters for respondents aged 25–49 (i.e., 1.66) by the number of respondents (age group 15–19 and 20–24). Originally the number of sisters was 716 for the age 15–19 years, and 852 for 20–24 years.

LTR = 15/1887 = 0.008, TFR = 4.6 for 3-previous years, and MM Ratio= 1-(1- LTR)^1/TFR^ = 175/100,000 LB (95%CI: 85, 263).

**Middle**

| **Age of respondents**  **in years** | **No of respondents** | **No. of sisters survived (>15 years)** | **No. of sisters died from all maternal causes** | **No. of pregnancy-related deaths** | **Adjustment**  **factors** | **Sister unit of exposure (E)** |
| --- | --- | --- | --- | --- | --- | --- |
|  |  | **(A)** | **(B)** | **(C)** | **(D)** | **(E=A*D)** |
| 15-19 | 638 | 1206* | 3 | 0 | 0.107 | 129 |
| 20-24 | 476 | 919^ | 19 | 3 | 0.206 | 189 |
| 25-29 | 546 | 1013 | 13 | 6 | 0.343 | 347 |
| 30-34 | 464 | 928 | 21 | 10 | 0.503 | 467 |
| 35-39 | 422 | 763 | 5 | 3 | 0.664 | 507 |
| 40-44 | 240 | 495 | 11 | 4 | 0.802 | 397 |
| 45-49 | 194 | 326 | 7 | 3 | 0.900 | 293 |
| **Total** | **2980** | **5650** | **79** | **29** |  | **2330** |

* Adjusted number of sisters by multiplying the average number of sisters for respondents aged 25–49 (i.e., 1.89) by the number of respondents (age group 15–19). Originally the number of sisters was 798 for 15–19 years.

^ No adjustment. As the number of sisters after adjustment was much less than the number of sisters reported, the actual number of sisters was used. Because, after adjustment, the number of sisters was 900 for the age 20–24 years.

LTR = 29/2330 = 0.013, TFR = 4.6 for 3-previous years, and MM Ratio = 1-(1- LTR)^1/TFR^ = 284/100,000 LB (95%CI: 181, 385).

**Fourth**

| **Age of respondents**  **in years** | **No of respondents** | **No. of sisters survived (>15 years)** | **No. of sisters died from all maternal causes** | **No. of pregnancy-related deaths** | **Adjustment**  **Factors** | **Sister unit of exposure (E)** |
| --- | --- | --- | --- | --- | --- | --- |
|  |  | **(A)** | **(B)** | **(C)** | **(D)** | **(E=A*D)** |
| 15-19 | 716 | 1411* | 15 | 5 | 0.107 | 151 |
| 20-24 | 513 | 1127^ | 12 | 3 | 0.206 | 232 |
| 25-29 | 510 | 1124 | 19 | 8 | 0.343 | 386 |
| 30-34 | 432 | 971 | 13 | 5 | 0.503 | 488 |
| 35-39 | 384 | 801 | 6 | 1 | 0.664 | 532 |
| 40-44 | 303 | 505 | 7 | 0 | 0.802 | 405 |
| 45-49 | 241 | 284 | 0 | 0 | 0.900 | 256 |
| **Total** | **3099** | **6223** | **72** | **22** |  | **2450** |

* Adjusted number of sisters by multiplying the average number of sisters for respondents aged 25–49 (i.e., 1.97) by the number of respondents (age group 15–19). Originally the number of sisters was 789 for 15–19 years.

^ No adjustment. As the number of sisters after adjustment was much less than the number of sisters reported, the actual number of sisters was used. Because, after adjustment, the number of sisters was 1011 for the age 20–24 years.

LTR = 22/2450 = 0.009, TFR = 4.6 for 3-prevous years, and MM Ratio = 1-(1- LTR)1^/TFR^ = 196/100,000 LB (95%CI: 115, 276).

**Highest**

| **Age of respondents**  **in years** | **No of respondents** | **No. of sisters survived (>15 years)** | **No. of sisters died from all maternal causes** | **No. of pregnancy-related deaths** | **Adjustment**  **factors** | **Sister unit of exposure (E)** |
| --- | --- | --- | --- | --- | --- | --- |
|  |  | **(A)** | **(B)** | **(C)** | **(D)** | **(E=A*D)** |
| 15-19 | 992 | 1944* | 23 | 0 | 0.107 | 208 |
| 20-24 | 782 | 1533* | 17 | 11 | 0.206 | 316 |
| 25-29 | 851 | 1462 | 7 | 1 | 0.343 | 501 |
| 30-34 | 599 | 1304 | 29 | 8 | 0.503 | 656 |
| 35-39 | 440 | 982 | 16 | 4 | 0.664 | 652 |
| 40-44 | 268 | 607 | 15 | 0 | 0.802 | 487 |
| 45-49 | 231 | 324 | 9 | 0 | 0.900 | 292 |
| **Total** | **4163** | **8156** | **116** | **24** |  | **3112** |

* Adjusted number of sisters by multiplying the average number of sisters for respondents aged 25–49 (i.e., 1.96) by the number of respondents (age group 15–19 and 20–24). Originally the number of sisters was 1080 for the age 15–19 years, and 1412 for 20–24 years. LTR = 24/3112 = 0.008, TFR = 4.6 for 3-previous years, and MM Ratio = 1-(1- LTR)^1/TFR^ =195/100,000 LB (95%CI: 107, 241).

**Sub-national administrative regions**

**Tigray**

| **Age of respondents**  **in years** | **No of respondents** | **No. of sisters survived (>15 years)** | **No. of sisters died from all maternal causes** | **No. of pregnancy-related deaths** | **Adjustment**  **factors** | **Sister unit of exposure (E)** |
| --- | --- | --- | --- | --- | --- | --- |
|  |  | **(A)** | **(B)** | **(C)** | **(D)** | **(E=A*D)** |
| 15-19 | 276 | 527* | 3 | 0 | 0.107 | 56 |
| 20-24 | 222 | 424* | 9 | 2 | 0.206 | 87 |
| 25-29 | 170 | 364 | 6 | 1 | 0.343 | 125 |
| 30-34 | 138 | 289 | 9 | 3 | 0.503 | 145 |
| 35-39 | 142 | 264 | 7 | 2 | 0.664 | 175 |
| 40-44 | 96 | 186 | 2 | 1 | 0.802 | 149 |
| 45-49 | 85 | 105 | 5 | 0 | 0.900 | 95 |
| **Total** | **1129** | **2159** | **41** | **9** |  | **833** |

*Adjusted number of sisters by multiplying the average number of sisters for respondents aged 25–49 (i.e., 1.91) by the number of respondents (age group 15–19 and 20–24). Originally the number of sisters was 281 for the age 15–19 years, and 312 for 20–24 years.

LTR = 9/833 = 0.011, TFR = 4.6 for 3-previous years, and MM Ratio = 1-(1- LTR)^1/TFR^ = 240/100,000 LB (95%CI: 85, 394).

**Afar**

| **Age of respondents**  **in years** | **No of respondents** | **No. of sisters survived (>15 years)** | **No. of sisters died from all maternal causes** | **No. of pregnancy-related deaths** | **Adjustment**  **factors** | **Sister unit of exposure (E)** |
| --- | --- | --- | --- | --- | --- | --- |
|  |  | **(A)** | **(B)** | **(C)** | **(D)** | **(E=A*D)** |
| 15-19 | 30 | 40* | 0 | 0 | 0.107 | 4 |
| 20-24 | 27 | 38^ | 0 | 0 | 0.206 | 8 |
| 25-29 | 26 | 34 | 0 | 0 | 0.343 | 12 |
| 30-34 | 18 | 27 | 0 | 0 | 0.503 | 14 |
| 35-39 | 13 | 17 | 0 | 0 | 0.664 | 11 |
| 40-44 | 8 | 11 | 0 | 0 | 0.802 | 9 |
| 45-49 | 8 | 7 | 0 | 0 | 0.900 | 6 |
| **Total** | **130** | **174** | **0** | **0** |  | **64** |

* Adjusted number of sisters by multiplying the average number of sisters for respondents aged 25–49 (i.e., 1.32) by the number of respondents (age group 15–19). Originally the number of sisters was 35 for 15–19 years.

^ No adjustment. As the number of sisters after adjustment was much less than the number of sisters reported, the actual number of sisters was used. Because, after adjustment, the number of sisters was 36 for the age 20–24 years.

LTR = 0/64 = 0, TFR = 4.6 for 3-previous years, and MM Ratio = 1-(1- LTR)^1/TFR^ = 0/100,000 LB (95%CI: 0, 0).

**Amhara**

| **Age of respondents**  **in years** | **No of respondents** | **No. of sisters survived (>15 years)** | **No. of sisters died from all maternal causes** | **No. of pregnancy-related deaths** | **Adjustment**  **factors** | **Sister unit of exposure (E)** |
| --- | --- | --- | --- | --- | --- | --- |
|  |  | **(A)** | **(B)** | **(C)** | **(D)** | **(E=A*D)** |
| 15-19 | 767 | 1404* | 15 | 0 | 0.107 | 150 |
| 20-24 | 615 | 1125* | 15 | 5 | 0.206 | 232 |
| 25-29 | 718 | 1193 | 5 | 0 | 0.343 | 409 |
| 30-34 | 529 | 1166 | 36 | 9 | 0.503 | 586 |
| 35-39 | 472 | 934 | 13 | 2 | 0.664 | 620 |
| 40-44 | 328 | 607 | 9 | 2 | 0.802 | 487 |
| 45-49 | 284 | 376 | 0 | 0 | 0.900 | 338 |
| **Total** | **3713** | **6805** | **93** | **18** |  | **2823** |

* Adjusted number of sisters by multiplying the average number of sisters for respondents aged 25–49 (i.e., 1.83) by the number of respondents (age group 15–19 and 20–24). Originally the number of sisters was 857 for the age 15–19 years, and 1082 for 20–24 years.

LTR = 18/2823 = 0.007, TFR = 4.6 for 3-previous years, and MM Ratio = 1-(1- LTR)^1/TFR^ = 153/100,000 LB (95%CI: 85, 222).

**Oromiya**

| **Age of respondents**  **in years** | **No of respondents** | **No. of sisters survived (>15 years)** | **No. of sisters died from all maternal causes** | **No. of pregnancy-related deaths** | **Adjustment**  **factors** | **Sister unit of exposure (E)** |
| --- | --- | --- | --- | --- | --- | --- |
|  |  | **(A)** | **(B)** | **(C)** | **(D)** | **(E=A*D)** |
| 15-19 | 1234 | 2809* | 31 | 8 | 0.107 | 301 |
| 20-24 | 995 | 2032^ | 41 | 17 | 0.206 | 419 |
| 25-29 | 1062 | 1934 | 40 | 17 | 0.343 | 663 |
| 30-34 | 924 | 1731 | 31 | 10 | 0.503 | 871 |
| 35-39 | 702 | 1303 | 8 | 3 | 0.664 | 865 |
| 40-44 | 428 | 800 | 11 | 0 | 0.802 | 642 |
| 45-49 | 356 | 450 | 12 | 3 | 0.900 | 405 |
| **Total** | **5701** | **11059** | **174** | **58** |  | **4165** |

* Adjusted number of sisters by multiplying the average number of sisters for respondents aged 25–49 (i.e., 1.79) by the number of respondents (age group 15–19). Originally the number of sisters was 1621 for 15–19 years.

^ No adjustment. As the number of sisters after adjustment was much less than the number of sisters reported, the actual number of sisters was used. Because, after adjustment, the number of sisters was 1781 for the age 20–24 years.

LTR = 58/4165 = 0.014, TFR = 4.6 for 3-previous years, and MM Ratio = 1-(1- LTR)^1/TFR^ = 306/100,000 LB (95%CI: 228, 380).

**Somali**

| **Age of respondents**  **in years** | **No of respondents** | **No. of sisters survived (>15 years)** | **No. of sisters died from all maternal causes** | **No. of pregnancy-related deaths** | **Adjustment**  **factors** | **Sister unit of exposure (E)** |
| --- | --- | --- | --- | --- | --- | --- |
|  |  | **(A)** | **(B)** | **(C)** | **(D)** | **(E=A*D)** |
| 15-19 | 105 | 214* | 4 | 1 | 0.107 | 23 |
| 20-24 | 81 | 185^ | 2 | 0 | 0.206 | 38 |
| 25-29 | 86 | 174 | 2 | 0 | 0.343 | 60 |
| 30-34 | 62 | 160 | 1 | 1 | 0.503 | 80 |
| 35-39 | 52 | 109 | 0 | 0 | 0.664 | 72 |
| 40-44 | 48 | 73 | 1 | 0 | 0.802 | 59 |
| 45-49 | 26 | 44 | 0 | 0 | 0.900 | 40 |
| **Total** | **460** | **959** | **10** | **2** |  | **372** |

* Adjusted number of sisters by multiplying the average number of sisters for respondents aged 25–49 (i.e., 2.04) by the number of respondents (age group 15–19). Originally the number of sisters was 157 for 15–19 years.

^ No adjustment. As the number of sisters after adjustment was much less than the number of sisters reported, the actual number of sisters was used. Because, after adjustment, the number of sisters was 165 for the age 20–24 years.

LTR = 2/372 = 0.006, TFR = 4.6 for 3-previous years, and MM Ratio = 1-(1- LTR)^1/TFR^ = 131/100,000 LB (95%CI: -40, 300).

**Beshngul Gumuz**

| **Age of respondents**  **in years** | **No of respondents** | **No. of sisters survived (>15 years)** | **No. of sisters died from all maternal causes** | **No. of pregnancy-related deaths** | **Adjustment**  **factors** | **Sister unit of exposure (E)** |
| --- | --- | --- | --- | --- | --- | --- |
|  |  | **(A)** | **(B)** | **(C)** | **(D)** | **(E=A*D)** |
| 15-19 | 34 | 62* | 0 | 0 | 0.107 | 7 |
| 20-24 | 32 | 58* | 0 | 0 | 0.206 | 12 |
| 25-29 | 28 | 52 | 0 | 0 | 0.343 | 18 |
| 30-34 | 23 | 50 | 0 | 0 | 0.503 | 25 |
| 35-39 | 22 | 33 | 0 | 0 | 0.664 | 22 |
| 40-44 | 13 | 22 | 0 | 0 | 0.802 | 18 |
| 45-49 | 8 | 14 | 0 | 0 | 0.900 | 13 |
| **Total** | **160** | **291** | 0 | 0 |  | **114** |

* Adjusted number of sisters by multiplying the average number of sisters for respondents aged 25–49 (i.e.,1.82) by the number of respondents (age group 15–19 and 20–24). Originally the number of sisters was 43 for the age 15–19 years, and 54 for 20–24 years.

LTR = 0/114 = 0, TFR = 4.6 for 3-previous years, and MM Ratio = 1-(1- LTR)^1/TFR^ = 0/100,000 LB (95%CI: 0, 0).

**SNNP**

| **Age of respondents**  **in years** | **No of respondents** | **No. of sisters survived (>15 years)** | **No. of sisters died from all maternal causes** | **No. of pregnancy-related deaths** | **Adjustment**  **factors** | **Sister unit of exposure (E)** |
| --- | --- | --- | --- | --- | --- | --- |
|  |  | **(A)** | **(B)** | **(C)** | **(D)** | **(E=A*D)** |
| 15-19 | 681 | 1308* | 15 | 1 | 0.107 | 140 |
| 20-24 | 571 | 1106^ | 16 | 0 | 0.206 | 228 |
| 25-29 | 666 | 1237 | 20 | 4 | 0.343 | 424 |
| 30-34 | 483 | 990 | 15 | 6 | 0.503 | 498 |
| 35-39 | 406 | 854 | 9 | 2 | 0.664 | 567 |
| 40-44 | 286 | 514 | 15 | 6 | 0.802 | 412 |
| 45-49 | 196 | 306 | 8 | 2 | 0.900 | 275 |
| **Total** | **3289** | **6315** | **98** | **21** |  | **2545** |

* Adjusted number of sisters by multiplying the average number of sisters for respondents aged 25–49 (i.e., 1.92) by the number of respondents (age group 15–19). Originally the number of sisters was 811 for 15–19 years.

^ No adjustment. As the number of sisters after adjustment was much less than the number of sisters reported, the actual number of sisters was used. Because, after adjustment, the number of sisters was 1096 for the age 20–24 years.

LTR = 21/2545 = 0.009, TFR = 4.6 for 3-previous years, and MM Ratio = 1-(1- LTR)^1/TFR^ = 196/100,000 LB (95%CI: 115, 276).

**Gambella**

| **Age of respondents**  **in years** | **No of respondents** | **No. of sisters survived (>15 years)** | **No. of sisters died from all maternal causes** | **No. of pregnancy-related deaths** | **Adjustment**  **factors** | **Sister unit of exposure (E)** |
| --- | --- | --- | --- | --- | --- | --- |
|  |  | **(A)** | **(B)** | **(C)** | **(D)** | **(E=A*D)** |
| 15-19 | 9 | 12* | 0 | 0 | 0.107 | 1 |
| 20-24 | 9 | 12* | 0 | 0 | 0.206 | 2 |
| 25-29 | 8 | 11 | 0 | 0 | 0.343 | 4 |
| 30-34 | 7 | 10 | 0 | 0 | 0.503 | 5 |
| 35-39 | 5 | 6 | 0 | 0 | 0.664 | 4 |
| 40-44 | 3 | 3 | 0 | 0 | 0.802 | 2 |
| 45-49 | 2 | 2 | 0 | 0 | 0.900 | 2 |
| **Total** | 43 | 56 | 0 | 0 |  | 21 |

* Adjusted number of sisters by multiplying the average number of sisters for respondents aged 25–49 (i.e., 1.28) by the number of respondents (age group 15–19 and 20–24). Originally the number of sisters was 8 for the age 15–19 years, and 12 for 20–24 years.

LTR = 0/21 = 0, TFR = 4.6 for 3-previous years, and MM Ratio = 1-(1- LTR)^1/TFR^ = 0/100,000 LB (95%CI: 0, 0).

**Harari**

| **Age of respondents**  **in years** | **No of respondents** | **No. of sisters survived (>15 years)** | **No. of sisters died from all maternal causes** | **No. of pregnancy-related deaths** | **Adjustment**  **Factors** | **Sister unit of exposure (E)** |
| --- | --- | --- | --- | --- | --- | --- |
|  |  | **(A)** | **(B)** | **(C)** | **(D)** | **(E=A*D)** |
| 15-19 | 8 | 13* | 0 | 0 | 0.107 | 1 |
| 20-24 | 8 | 15^ | 0 | 0 | 0.206 | 3 |
| 25-29 | 7 | 12 | 0 | 0 | 0.343 | 4 |
| 30-34 | 6 | 10 | 0 | 0 | 0.503 | 5 |
| 35-39 | 5 | 8 | 0 | 0 | 0.664 | 5 |
| 40-44 | 4 | 4 | 0 | 0 | 0.802 | 3 |
| 45-49 | 2 | 4 | 0 | 0 | 0.900 | 4 |
| **Total** | 40 | 66 | 0 | 0 |  | 26 |

* Adjusted number of sisters by multiplying the average number of sisters for respondents aged 25–49 (i.e., 1.58) by the number of respondents (age group 15–19). Originally the number of sisters was 9 for 15–19 years.

^ No adjustment. As the number of sisters after adjustment was much less than the number of sisters reported, the actual number of sisters was used. Because, after adjustment, the number of sisters was 13 for the age 20–24 years.

LTR = 0/26 = 0, TFR = 4.6 for 3-previous years, and MM Ratio = 1-(1- LTR)^1/TFR^ = 0/100,000 LB (95%CI: 0, 0).

**Addis Ababa**

| **Age of respondents**  **in years** | **No of respondents** | **No. of sisters survived (>15 years)** | **No. of sisters died from all maternal causes** | **No. of pregnancy-related deaths** | **Adjustment**  **Factors** | **Sister unit of exposure (E)** |
| --- | --- | --- | --- | --- | --- | --- |
|  |  | **(A)** | **(B)** | **(C)** | **(D)** | **(E=A*D)** |
| 15-19 | 217 | 393* | 4 | 0 | 0.107 | 42 |
| 20-24 | 186 | 337* | 2 | 0 | 0.206 | 69 |
| 25-29 | 168 | 286 | 2 | 0 | 0.343 | 98 |
| 30-34 | 142 | 257 | 7 | 2 | 0.503 | 129 |
| 35-39 | 104 | 207 | 4 | 1 | 0.664 | 137 |
| 40-44 | 69 | 133 | 3 | 0 | 0.802 | 107 |
| 45-49 | 45 | 74 | 2 | 0 | 0.900 | 67 |
| **Total** | 931 | 1687 | **24** | **3** |  | 650 |

* Adjusted number of sisters by multiplying the average number of sisters for respondents aged 25–49 (i.e., 1.81) by the number of respondents (age group 15–19 and 20–24). Originally the number of sisters was 215 for the age 15–19 years, and 265 for 20–24 years.

LTR = 3/650 = 0.005, TFR = 4.6 for 3-previous years, and MM Ratio = 1-(1- LTR)^1/TFR^ = 109/100,000 LB (95%CI: -11, 228).

**Dire Dawa**

| **Age of respondents**  **in years** | **No of respondents** | **No. of sisters survived (>15 years)** | **No. of sisters died from all maternal causes** | **No. of pregnancy-related deaths** | **Adjustment**  **Factors** | **Sister unit of exposure (E)** |
| --- | --- | --- | --- | --- | --- | --- |
|  |  | **(A)** | **(B)** | **(C)** | **(D)** | **(E=A*D)** |
| 15-19 | 20 | 35* | 0 | 0 | 0.107 | 4 |
| 20-24 | 17 | 27^ | 0 | 0 | 0.206 | 6 |
| 25-29 | 19 | 32 | 0 | 0 | 0.343 | 11 |
| 30-34 | 14 | 25 | 0 | 0 | 0.503 | 13 |
| 35-39 | 9 | 20 | 0 | 0 | 0.664 | 13 |
| 40-44 | 7 | 12 | 0 | 0 | 0.802 | 10 |
| 45-49 | 5 | 5 | 0 | 0 | 0.900 | 5 |
| **Total** | **91** | **156** | 0 | 0 |  | **60** |

* Adjusted number of sisters by multiplying the average number of sisters for respondents aged 25–49 (i.e., 1.74) by the number of respondents (age group 15–19). Originally the number of sisters was 24 for 15–19 years.

^ No adjustment. As the number of sisters after adjustment was much less than the number of sisters reported, the actual number of sisters was used. Because, after adjustment, the number of sisters was 30 for the age 20–24 years.

LTR = 0/60 = 0, TFR = 4.6 for 3-previous years, and MM Ratio = 1-(1- LTR)^1/TFR^ = 0/100,000 LB (95%CI: 0, 0).
